# The histone variant H2A.X is a regulator of the epithelial–mesenchymal transition

**DOI:** 10.1038/ncomms10711

**Published:** 2016-02-15

**Authors:** Urbain Weyemi, Christophe E. Redon, Rohini Choudhuri, Towqir Aziz, Daisuke Maeda, Myriem Boufraqech, Palak R. Parekh, Taresh K. Sethi, Manjula Kasoji, Natalie Abrams, Anand Merchant, Vinodh N. Rajapakse, William M. Bonner

**Affiliations:** 1 Developmental Therapeutics Branch, Laboratory of Molecular Pharmacology, National Cancer Institute, National Institutes of Health, 37 Convent Drive, Bethesda, Maryland 20892, USA; 2 Endocrine Oncology Branch, Center for Cancer Research, National Cancer Institute, National Institutes of Health, 9000 Rockville Pike, Bethesda, Maryland 20892, USA; 3 Center for Cancer Research Collaborative Bioinformatics Resource, National Cancer Institute, 37 Convent Drive, Bethesda, Maryland 20892, USA

## Abstract

The epithelial–mesenchymal transition (EMT), considered essential for metastatic cancer, has been a focus of much research, but important questions remain. Here, we show that silencing or removing H2A.X, a histone H2A variant involved in cellular DNA repair and robust growth, induces mesenchymal-like characteristics including activation of EMT transcription factors, Slug and ZEB1, in HCT116 human colon cancer cells. Ectopic H2A.X re-expression partially reverses these changes, as does silencing Slug and ZEB1. In an experimental metastasis model, the HCT116 parental and H2A.X-null cells exhibit a similar metastatic behaviour, but the cells with re-expressed H2A.X are substantially more metastatic. We surmise that H2A.X re-expression leads to partial EMT reversal and increases robustness in the HCT116 cells, permitting them to both form tumours and to metastasize. In a human adenocarcinoma panel, H2A.X levels correlate inversely with Slug and ZEB1 levels. Together, these results point to H2A.X as a regulator of EMT.

## Abstract (web summary)

The histone H2A variants are involved in DNA repair, gene regulation and cancer development. In this study, the authors unravel an additional role for H2A.X in the regulation of mesenchymal-like traits and activation of the EMT transcription factors, Slug and ZEB1, in colon cancer cells.

## Graphical abstract

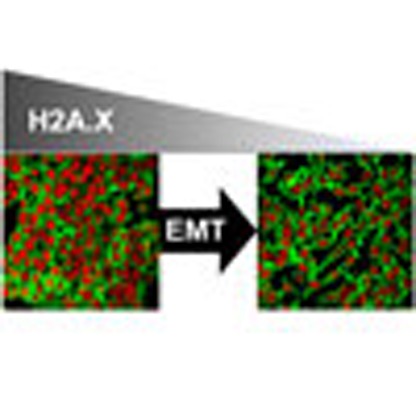

The spread of cancerous cells to distant organs is a longstanding obstacle to successful cancer treatment. The process by which cells from primary tumours acquire the ability to form distant tumours involves the loss of cell-to-cell adhesion as well as the disruption of the apicobasal polarity, and the transition to a cell type with a more spindle-like morphology^1^. Such changes enable the cells to invade the extracellular matrix^2^. This reversible physiological process is known as the epithelial–mesenchymal transition (EMT or MET in reverse). The molecular mechanisms underlying EMT include decreased expression of a set of epithelial genes with the concomitant activation of a set of mesenchymal genes, the expression of matrix metalloproteinases markers and the formation of lamellipodia, filopodia and invadopodia^3^,^4^. At distant sites, some mesenchymal cells may be involved in the establishment of tumours^2^,^5^,^6^ in a process thought to require at least partial re-acquisition of epithelial characteristics.

Changes in chromatin configuration have emerged as key to EMT-related transcription factor regulation^1^,^7^,^8^,^9^,^10^, but some of these changes still call for further characterization. While the four nucleosome histone families provide equal numbers of molecules to the nucleosome, several of the families include multiple variants, whose stoichiometry can vary due to cell type and growth state among other factors^11^,^12^,^13^. Altered expression of variants in several histone families, including H2A has been associated with cancer^14^. Recently, it has been reported that histone H2A variant macroH2A is a critical component of chromatin that suppresses the progression of melanoma^15^.

Histone H2A.X also belongs to the histone H2A family. Like other histone variants, H2A.X is highly conserved among species and achieves critical cellular functions beyond those fulfilled by canonical H2As. H2A.X plays essential roles in DNA double-strand break repair and genome stability, and is classified as a tumour suppressor. As with other H2A variants, the relative amount of H2A.X varies among cell lines^16^,^17^. How this variation may affect the transcription regulation of other genes remains poorly investigated.

While comparing growth characteristics of H2A.X-null cells with parental lines, we observed that the null cells exhibited elevated levels of migration and invasion, characteristic of the EMT transition. Given these observations and the increasing evidence for the role of other histone variants in the regulation of gene transcription^18^,^19^ and cancer progression^16^,^20^,^21^, we hypothesized that the downregulation of histone variant H2A.X may contribute to the alteration of chromatin configuration and induce changes in cancer gene expression.

Our novel findings provide evidence that H2A.X depletion activates the EMT programme in at least some human colorectal adenocarcinoma cells. The loss of H2A.X was strongly correlated with the EMT-inducing transcription factors Slug and ZEB1 in these cells. These correlations were substantiated by the observations that the silencing of Slug and ZEB1 abrogated the mesenchymal phenotype exhibited by H2A.X-depleted cells. Most importantly, restored expression of H2A.X at least partially reversed the EMT programme induced by H2A.X loss. H2A.X-deficient cells are proliferation defective, and sensitive to environmental and genotoxic stresses^20^,^22^; characteristics which may counteract their increased invasiveness and account for the lack of enhanced metastasis *in vivo* compared with parental cells. However, in the H2A.X revertants, proliferation is enhanced, but sufficient invasiveness may remain to result in elevated numbers of metastatic lung foci. Taken together, our results demonstrate that H2A.X may be a novel regulator of the EMT programme and suggest a role for H2A.X in cancer progression and metastasis.

## Results and Discussion

### H2A.X regulates EMT and colon cancer metastasis signalling

**Figure 1:**
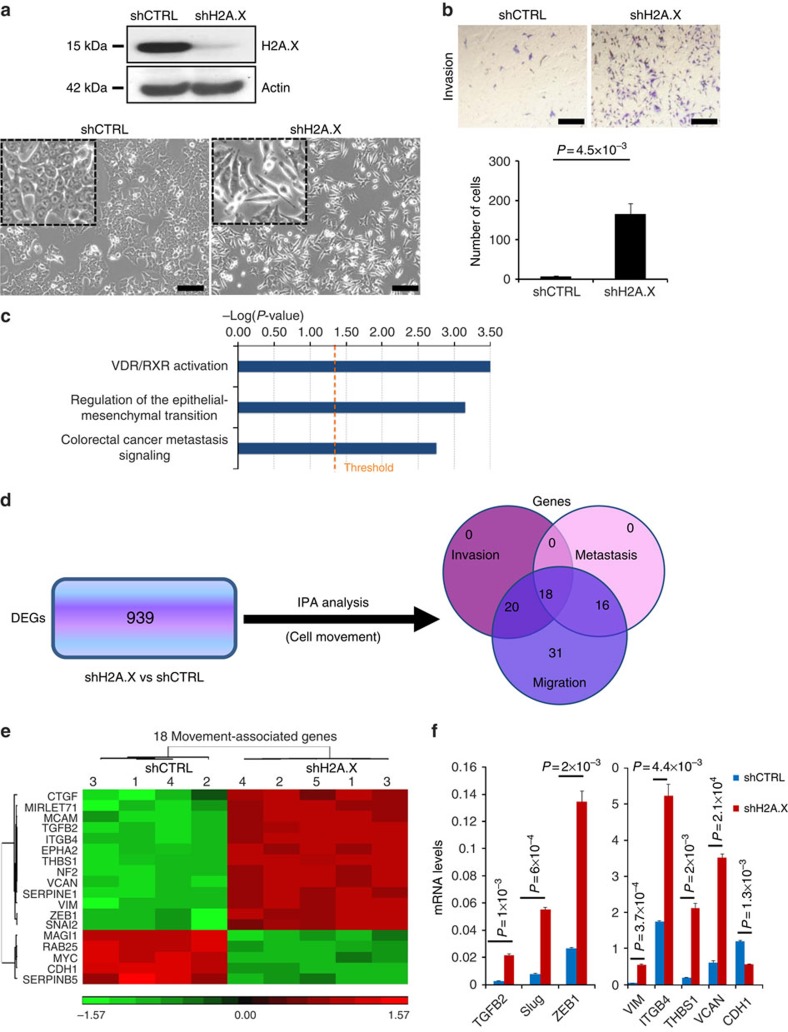
H2A.X is a key player in the regulation of EMT and in colon cancer metastasis signalling. HCT116 cells were infected with shRNAs against H2A.X (shH2A.X) or scrambled sequences (shCTRL). (**a**,**b**) Compared with controls, shH2A.X-infected HCT116 cells exhibited decreased H2A.X protein levels (**a**, top, immunoblot), mesenchymal-like morphology (**a**, bottom, photomicrographs, scale bar, 100 μm) and increased invasion in transwell invasion assays (**b**, top, photomicrograph, scale bar, 100 μm; bottom, quantification). Error bars represent the means±s.d. of three independent experiments. Statistical significance was determined by a two-tailed, unpaired Student's *t*-test. (**c**) Ingenuity Pathway Analysis (IPA) revealed differentially expressed genes (DEGs) in three canonical pathways. Blue bars represent the top three canonical pathways overrepresented in the DEGs. The height of the bar represents the *P* value. The yellow line indicates the −log (*P* value) threshold of significance (1.3), which corresponds to *P* value of 0.05. Statistical significance was determined by Fisher's test. (**d**) Venn diagram of DEGs assigned to the invasion, migration and metastasis pathways using IPA. DEGs were generated using Partek Genomics Suite software, version 6.6. (**e**) Heat map of the 18 DEGs common to the invasion, migration and metastasis pathways. The numbering refers to independent replicates for either shCTRL sample or shH2A.X sample. (**f**) Verification of differential expression of genes by real-time PCR). Expression values are relative fold change for gene transcripts normalized to 18S RNA (gene/18S ratio). Error bars represent s.d. (*n*=3). Statistical significance was determined by a two-tailed, unpaired Student's *t*-test.

We observed that when cultures of the human colon cancer line HCT116 were made deficient in histone H2A.X, they lost their epithelial shape, became more mesenchymal-like (Fig. 1a), and more invasive (Fig. 1b). These findings suggested a possible role for histone H2A.X in EMT. We then performed a genome-wide differential gene expression analysis comparing H2A.X-deficient and control HCT116 cells (Full data available at http://www.ncbi.nlm.nih.gov/geo/query/acc.cgi?token=mdcbmqikhdcvtsp&acc=GSE75444). Pathway analysis of the differentially expressed genes using Ingenuity Pathway Analysis (IPA) revealed a significant enrichment for multiple signalling pathways involved in cancer, with the EMT and the colorectal cancer metastasis signalling pathways ranking among the top three (Fig. 1c). The output from IPA using the 939 differentially expressed genes revealed 85 genes involved in cell movement (Fig. 1d.). Of these, 18 genes were common to three cell movement-associated phenotypes, including cell invasion, migration and metastasis (Fig. 1d); 13 genes known to be essential for mesenchymal traits increased in expression, while 5 epithelial genes exhibited decreased expression levels (Fig. 1e). Real-time PCR (RT–PCR) confirmed that silencing H2A.X promoted greatly increased expression of several key mesenchymal genes, including SNAI2 or Slug, ZEB1, vimentin (VIM), THBS1, versican (VCAN) and TGFB2, coupled with decreased expression of a key epithelial gene, CDH1 (Fig. 1f). Two of these genes, Slug and ZEB1, encode EMT-inducing transcription factors. Thus, our results point to Slug and ZEB1 as key EMT-related transcription factors regulated by H2A.X in colon cancer cells.

One possible explanation for these results is that the observed phenomena are not due specifically to H2A.X removal, but to a disruption in the orderly chromatin structure, which is manifested by EMT-like changes. If this is the case, then removal of other H2A variants might lead to the same EMT-like changes. One variant, macroH2A, has been reported to be involved in cancer progression and metastasis^15^, and EMT is considered essential for cancer metastasis^1^,^23^. However, when we investigated whether macroH2A.1 or H2A.Z downregulation in HCT116 cells led to EMT-like changes, little, if any, change in Slug or ZEB1 or the other genes examined was found (Supplementary Fig. 1a–d). Likewise, to investigate whether any transient changes in the pool of the nucleosomal H2A.X might result in the activation of EMT, we conducted protein- and transcript-level analysis of EMT markers in cells overexpressing H2A.X for 5 days. Data indicate that H2A.X overexpression did not substantially affect the expression level of the EMT markers examined (Supplementary Fig. 2a,b), whereas a slight activation of the EMT programme was observed in cells transiently silenced for H2A.X within the same period of time (Supplementary Fig. 2c). Thus, we conclude that EMT programme initiation in HCT116 cells can likely be attributed specifically to H2A.X silencing.

### Slug and ZEB1 mediate H2A.X loss-induced EMT

The EMT programme is orchestrated by a set of pleiotropically acting transcription factors, including Snail1/2 (refs 24, 25), ZEB1/2 (refs 26, 27), Twist1/2 (refs 28, 29), FOXC2 (ref. 30), among others^3^. It is widely accepted that the E-box-binding zinc finger transcription factors Slug and ZEB1 contribute to the EMT in primary epithelial cancers^31^; and a Slug-driven transcriptional activation of ZEB1 has been proposed for a cooperative regulation of the EMT programme in melanoma^32^.

**Figure 2:**
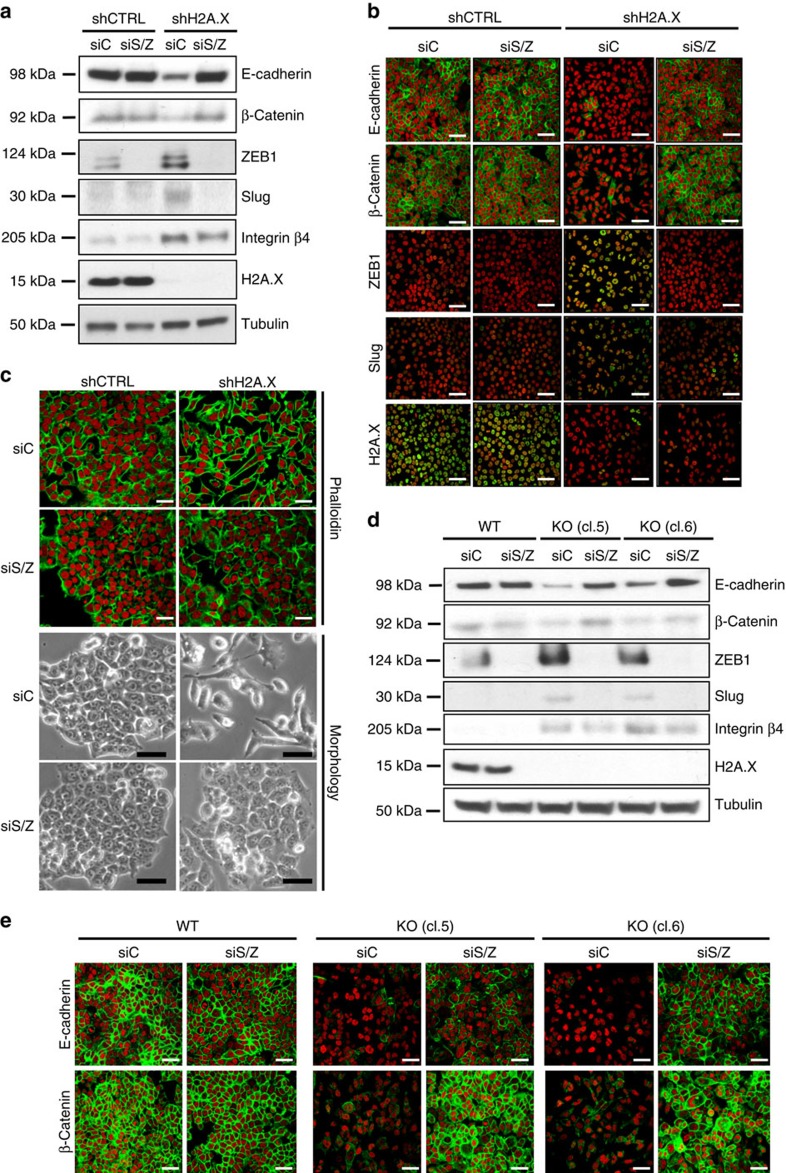
Downregulation of the transcription factors Slug and ZEB1 reverses the EMT programme induced by H2A.X deficiency. (**a**) Control cells (shCTRL) and cells silenced for H2A.X (shH2A.X) were transfected for 3 days with control siRNA (siC) or with a pool of siSLUG and siZEB1 (siS/Z). Immunoblot analysis of EMT markers was performed using tubulin as loading control. (**b**) Staining of E-cadherin and β-catenin by immunofluorescence in HCT116 control cells (shCTRL) and H2A.X-deficient cells (shH2A.X) transfected for 3 days with control siRNA (siC) or a pool of siRNAs against SLUG and ZEB1 (siS/Z). Nuclei were counterstained with propidium iodide (red); scale bars, 20 μm. (**c**) Top panel, analysis of cell phenotypic changes through the staining of F-actin (phalloidin) by immunofluorescence using conditions described in **b**; scale bars, 20 μm. Bottom panel, photomicrographs of cells; scale bar, 100 μm.(**d**) Immunoblot analysis of EMT markers in HCT116 parental cells (WT) and H2A.X knockout cells (KO) transfected for 3 days with siRNA control (siC) or with a pool of siSLUG and siZEB1 (siS/Z) with tubulin-loading controls. H2A.X knockout cells were generated using CRISPR/Cas9 system for precise deletion of the H2A.X gene. cl.5, clone #5; and cl.6, clone #6. (**e**) Immunofluorescence assays showing E-cadherin and β-catenin staining. Cells were treated as in **d**. Nuclei were counterstained in red with propidium iodide; scale bars, 20 μm.

Thus, the finding that H2A.X-deficient cells exhibited substantially increased Slug and ZEB1 expression raised the question of whether these transcription factors mediated H2A.X-regulated EMT. This was examined utilizing siRNAs to silence Slug and ZEB1 in the H2A.X-deficient cells. Expression of epithelial proteins including E-cadherin and β-catenin returned to near control levels as measured by immunoblotting (Fig. 2a) and immunofluorescence (Fig. 2b). However, although Slug and ZEB1 appeared to be fully silenced (Fig. 2a), and although the expression levels of the three examined mesenchymal genes, VIM, VCAN and integrin beta-4 (ITGB4), were decreased to varying degrees, they remained well above the values in the control cells (Fig. 2a and Supplementary Fig. 3). Independent silencing of Slug and ZEB1 suggested that these transcription factors may cooperate to regulate EMT in H2A.X-deficient cells, as the co-silencing of Slug and ZEB1 resulted in a greater reversal of the expression of the examined EMT markers than silencing each separately (Supplementary Fig. 4). In addition, silencing of ZEB1 alone seemed to partially affect the transcript level of Slug, further suggesting a link between Slug and ZEB1. Importantly, however, while silencing Slug and ZEB1 failed to lower the expression of mesenchymal factors to control levels, it was sufficient to restore the epithelial phenotype of H2A.X-deficient HCT116 cells (Fig. 2c). These observations indicate that the Slug and ZEB1 transcription factors are essential in mediating the EMT phenotype driven by H2A.X loss.

To confirm these observations, we generated H2A.X knockout HCT116 cells using the CRISPR/Cas9 procedure^33^. These H2A.X knockout HCT116 cells exhibited greatly increased expression of the mesenchymal genes including Slug, ZEB1, VIM, THBS1, VCAN and TGFB2, coupled with decreased expression of the epithelial gene CDH1 (Supplementary Fig. 5a), confirming our results using short hairpin RNA. H2A.X-null (H2A.X-KO) cells exhibited decreased protein levels of E-cadherin and β-catenin, coupled with increased ITGB4 levels similar to those found in the shH2A.X HCT116 cultures (Fig. 2d). Likewise, H2A.X depletion using CRISPR/Cas9 system in an additional colorectal cancer cell line, HCT15, resulted in the induction of the EMT programme and enhanced invasion, further confirming a broader effect of H2AX loss (Supplementary Fig. 5b–e). In addition, these results show that decreasing Slug and ZEB1 expression led to a partial reversal of the EMT induced by H2A.X removal, with the epithelial markers E-cadherin and β-catenin returning to near control levels but without large decreases of the mesenchymal marker ITGB4 (Fig. 2d,e and Supplementary Fig. 6).

As H2A.X is primarily known for its role in DNA damage repair^16^,^34^, the observation that H2A.X appeared to affect the EMT raises the question as to whether H2A.X directly influences the transcriptional regulation of Slug and ZEB1 or whether accumulation of DNA damage resulting from H2A.X deficiency indirectly activates Slug and ZEB1, perhaps through DNA damage pathway kinases such as ATR and ATM. Recently, ATM-mediated stabilization of ZEB1 was reported to link EMT to radioresistance *in vivo* as well as *in vitro*^35^. However, inactivation of ATM with KU55933 for 3 days in HCT116 cells with downregulated H2A.X did not result in altered ZEB1 protein levels (Supplementary Fig. 7), suggesting that H2A.X-deficiency-induced ZEB1 accumulation most likely involves a different mechanism.

**Figure 3:**
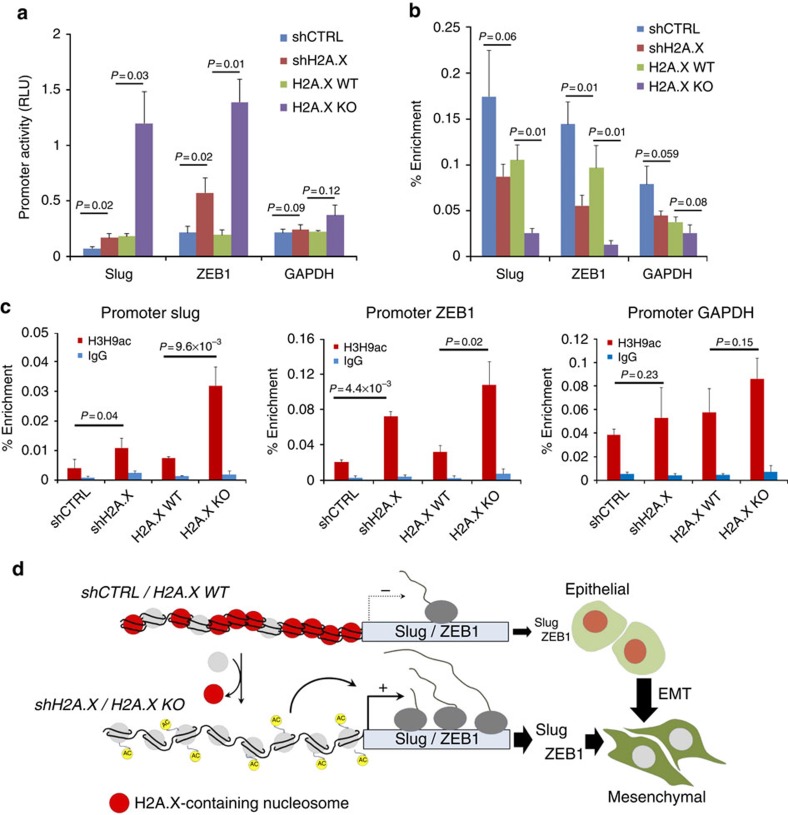
H2A.X removal from HCT116 cells leads to enhanced transcriptional activity of Slug and ZEB1. (**a**) H2A.X depletion enhanced the promoter activity of Slug and ZEB1, but not GAPDH. Slug, ZEB1 and GAPDH promoter activities were accessed by luciferase reporter assay in control cells (shCTRL), in cells silenced for H2A.X (shH2A.X), in parental cells (H2A.X WT) and in H2A.X knockout cells (H2A.X KO). Error bars represent the s.e.m. (*n*=3). Statistical significance was determined by a two-tailed, unpaired Student's *t*-test. (**b**) H2A.X binding to Slug and ZEB1 promoter using chromatin immunoprecipitation (ChIP) assay. Chromatin from control cells (shCTRL) and cells silenced for H2A.X (shH2A.X) or parental cells (H2A.X WT) and H2A.X knockout cells (H2A.X KO) was immunoprecipitated with anti-H2A.X antibody. The purified DNA was analysed by real-time PCR using primers amplifying across Slug, ZEB1 and GAPDH promoters. Results are presented as percentage of total input DNA precipitated. GAPDH promoter serves as an internal control. Error bars represent the s.e.m. (*n*=3). Statistical significance was determined by a two-tailed, unpaired Student's *t*-test (**c**) H2A.X deletion enhances the enrichment of H3K9ac to Slug and ZEB1 promoters. Cells used in **b** were processed for ChIP using anti-H3K9ac antibody. GAPDH promoter serves as an internal control. Error bars represent the s.e.m. (*n*=3). Statistical significance was determined by a two-tailed, unpaired Student's *t*-test. (**d**) Hypothetical model for the role of H2A.X in the transcriptional regulation of Slug and ZEB1 during EMT in colon cancer cells. H2A.X removal from the nucleosome leads to enhanced enrichment of active chromatin marks (H3K9ac) within the promoters of Slug and ZEB1. This chromatin configuration enables the transcriptional activation of Slug and ZEB1. Elevated levels of Slug and ZEB1 are key in mediating the expression of several EMT-related genes.

The effect of H2A.X on Slug and ZEB1 promoter activities was found to be a 2.44- and 2.70-fold activation, respectively, using shH2A.X, and a corresponding 6.44- and 7.24-fold activation with the H2A.X knockout (Fig. 3a). By contrast, the promoter activity of GAPDH, a gene known not to be involved in EMT, remained relatively unchanged. However, H2A.X overexpression did not lead to changes in Slug and ZEB1 promoter activity (Supplementary Fig. 8a,b). Chromatin immunoprecipitation (ChIP) of H2A.X and H3K9ac, a histone mark of active promoters (H3K9ac)^36^,^37^,^38^, revealed that reduction of H2A.X in the Slug and ZEB1 promoters (Fig. 3b) was strongly correlated with an accumulation of H3K9ac at the same locus (Fig. 3c). Similar results were obtained with an additional active transcription mark (H3K27ac)^39^,^40^ (Supplementary Fig. 8c). Thus, we surmise that H2A.X removal from the Slug and ZEB1 promoters may enhance their transcriptional activation through chromatin relaxation, subsequently leading to induction of the EMT programme (Fig. 3d). However, how this chromatin relaxation specifically occurs in EMT-related transcription factors loci still remains unclear.

### Restored expression of H2A.X induces partial EMT reversal

**Figure 4:**
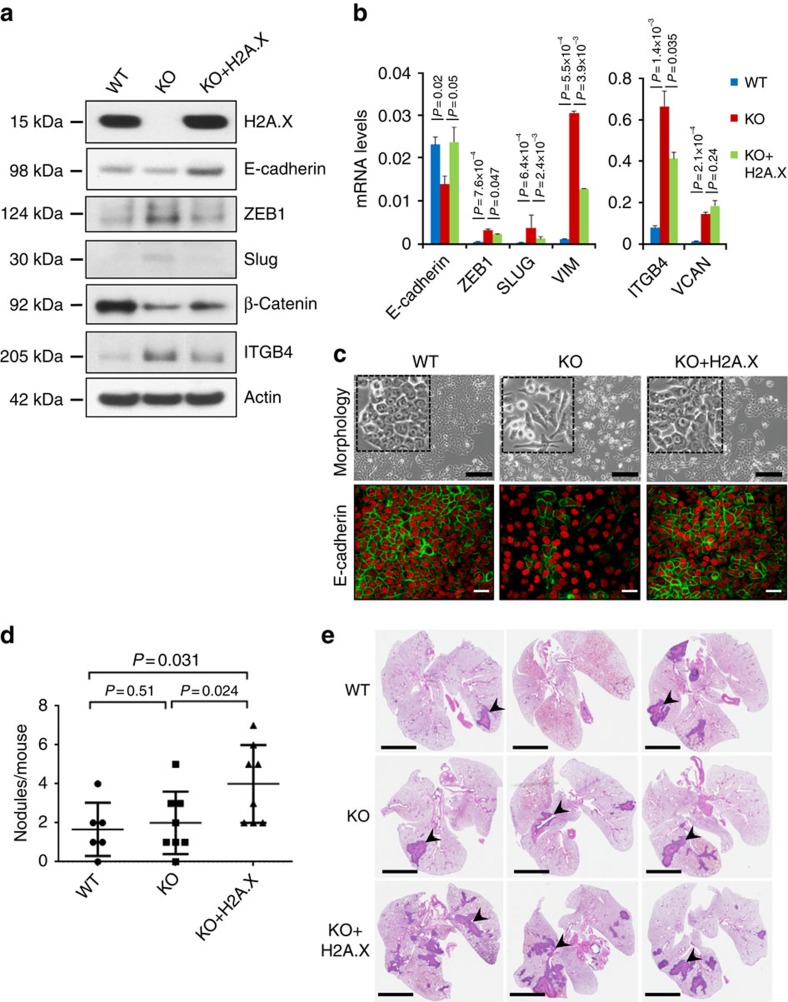
H2A.X re-expression inhibits EMT and promotes metastatic colonization in the lung. (**a**) Immunoblot analysis of EMT markers in HCT116 parental cells (WT), H2A.X knockout cells (KO) and H2A.X knockout cells in which H2A.X expression was restored (KO+H2A.X), utilizing actin as a loading control. (**b**) HCT116 parental cells (WT), H2A.X knockout cells (KO) and revertant cells (KO+H2A.X) were analysed for transcripts levels of EMT markers by real-time PCR. Expression values for E-cadherin (CDH1), ZEB1, Slug, Vimentin (VIM), integrin beta 4 (ITGB4) and versican (VCAN) were normalized to 18S RNA (gene/18S ratio). Error bars represent the s.e. (*n*=3). Statistical significance was determined by a two-tailed, unpaired Student's *t*-test. The experiments were repeated three times. (**c**). Immunostaining of the epithelial marker E-cadherin. Nuclei were counterstained in red with propidium iodide; scale bars, 20 μm. Photomicrographs of cells are shown (top panel); scale bar, 100 μm. (**d**) Tail vein injections of HCT116 parental cells (WT) and H2A.X knockout cells (KO) showed similar numbers of lung metastatic foci 4 weeks post injection. Ectopic expression of H2A.X (KO+H2A.X) resulted in a 2.5-fold increase in lung macroscopic metastases. (**e**) Representative haematoxylin- and eosin-stained lung sections from mice injected with HCT116 parental cells (WT), H2A.X knockout cells (KO) and H2A.X knockout cells in which H2A.X expression was restored (KO+H2A.X). Black arrows indicate some macroscopic lung nodules. Scale bars, 1 mm. Statistical significance was determined by a two-tailed, unpaired Student's *t*-test.

H2A.X expression was restored in the knockout HCT116 cells to examine whether the characteristics of the parental HCT116 cells would be restored. Revertant clones with parental H2A.X levels exhibited growth rates similar to those observed in the parental cells, while clones with lower H2A.X levels exhibited slower proliferation rates (Supplementary Fig. 9a–c). Interestingly, however, a quantitative assessment of key EMT protein and transcript levels revealed that the re-expression of normal levels of H2A.X did not completely restore the EMT markers to their original levels. Epithelial markers, including E-cadherin and β-catenin, increased in expression to levels close to those in the parental cells. Mesenchymal markers, including Slug, ZEB1 and ITGB4, decreased in expression, but the levels of these markers remained substantially higher than the parental levels (Fig. 4a,b). These findings are consistent with the revertants exhibiting a morphology intermediate between the H2A.X KO and H2A.X parental cells (Fig. 4c and Supplementary Fig. 9d).

### H2A.X re-expression promotes metastatic colonization

Since cancer metastasis may involve the ability to undergo both EMT and MET^2^,^3^, cells that have both epithelial and mesenchymal characteristics may have increased metastatic potential. To investigate how the presence, removal and reintroduction of H2A.X impacts the metastatic colonization potential of cancer cells, we injected parental, H2A.X knockout and H2A.X revertant cells into the tail veins of immuno-compromised mice and analysed the number of macroscopic lung metastases after 4 weeks (see ‘Methods' section). While the parental and knockout cells resulted in a similar number of metastatic lung foci (Fig. 4d,e), the cells with restored expression of H2A.X resulted in a 2.5-fold increase in the number of macroscopic metastases (Fig. 4d,e).

**Figure 5:**
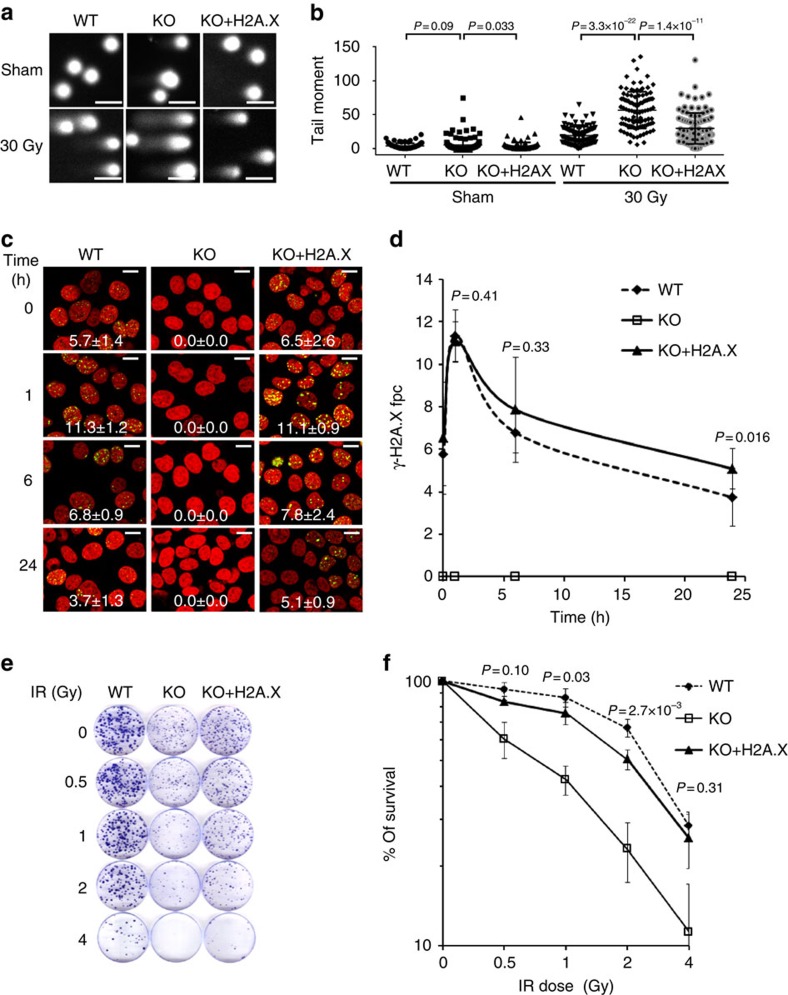
Ectopic expression of H2A.X enables efficient DNA repair and protects cells from genotoxic stress. (**a**,**b**) Alkaline comet assay of HCT116 parental cells (WT), H2A.X knockout cells (KO) and revertant cells (KO+H2A.X) 3 h after exposure to 30 Gy of ionizing radiation. (**a**) Representative images, scale bars, 100 μm; (**b**) Quantification of comet tail moments. Brackets indicate significance of differences. Error bars represent the s.e.m. (*n*=50). Statistical significance was determined by a two-tailed, unpaired Student's *t*-test.(**c**,**d**) HCT116 cell cultures (WT, KO and KO+H2A.X) were exposed to 1 Gy of ionizing radiation. DNA damage levels were analysed by counting γ-H2A.X foci (green) in nuclei counterstained for DNA (red). Scale bars, 20 μm. Statistical significance was determined by a two-tailed, unpaired Student's *t*-test. The experiments were repeated three times. (**c**) Representative images, × 40 magnification (**d**) Quantification of γ-H2A.X foci per cell. Data are means±s.d.; *n*=3. Fpc is foci per cell. Statistical significance was determined by a two-tailed, unpaired Student's *t*-test. (**e**,**f**) HCT116 cell cultures (WT, KO and KO+H2A.X) were exposed to increasing doses of ionizing radiation, and incubated at 37 °C for 15 days. Colonies stained with Coomassie blue were counted for survival estimation. (**e**) Representative images. (**f**) Colony survival. Data are means±s.d.; *n*=3. Statistical significance was determined by a two-tailed, unpaired Student's *t*-test.

These results indicate that re-introduction of H2A.X into HCT116 H2A.X-null cells induced an incomplete MET, and enhanced proliferative ability coupled with some retained mesenchymal characteristics. These observations are consistent with published reports supporting the requirement for MET and cell proliferation in establishing metastatic colonization^28^,^41^,^42^,^43^,^44^. When the H2A.X revertant cells were exposed to ionizing radiation, they exhibited levels of DNA damage (Fig. 5a,b), DNA repair capacity (Fig. 5c,d and Supplementary Fig. 10) and cell survival (Fig. 5e,f) similar to those of the parental cells, observations consistent with proposal that the survival of mesenchymal cells in the circulation requires resistance to environmental and genotoxic stresses^1^,^45^. Collectively, our observations suggest that restoration of H2A.X endows the H2A.X knockout cells with enhanced survival ability while still permitting some mesenchymal characteristics, thus leading to higher potential for metastatic colonization.

One intriguing issue is whether the DNA repair-associated motif of H2A.X, phosphorylatable Ser139 residue, is required for the ability to induce EMT reversal. To test this, we generated H2A.X knockout cells expressing a mutant H2A.X (H2A.X S139A) that is unable to detect DNA damage and is strongly associated with defective cell proliferation (Supplementary Fig. 11a–c). Surprisingly, the EMT reversal is impaired in H2A.X mutant revertants, just as in the H2A.X-null cells (Supplementary Fig. 11d, e). These results suggest that the DNA repair function of H2A.X may be required for its regulation of EMT-related genes. However, how S139 phosphorylation may control the direct transcriptional regulation of genes to which H2A.X-containng nucleosomes are bound still remains unclear.

Several clinical studies have pointed out that metastases arising from carcinomas exhibit a well- differentiated epithelial phenotype^46^,^47^. Also, in an experimental metastasis model, both primary tumours and metastatic nodules were found to express similar levels of key EMT markers^28^. In line with these findings, we compared the transcript levels of Slug, ZEB1 and H2A.X in a panel of 18 primary colorectal cancers and 18 matched liver metastases, using the publicly available data sets from Gene Expression Omnibus, GEO (*GSE14297*; ref. 48). The data indicate that both primary tumours and metastatic nodules displayed similar levels of Slug, ZEB1 and H2A.X (Supplementary Fig. 12), further supporting the idea that EMT markers are similar in the metastases and their primary carcinomas. Furthermore, Slug, ZEB1 and H2A.X display similar transcript levels in normal and primary tumour tissues from the same data sets, suggesting that these genes are not involved in the early stages of tumorigenesis. However, as the panel is very limited with only seven specimens from normal colon tissues, follow-up experiments with a large cohort will be needed.

### H2A.X correlates with Slug and ZEB1 in human adenocarcinoma

**Figure 6:**
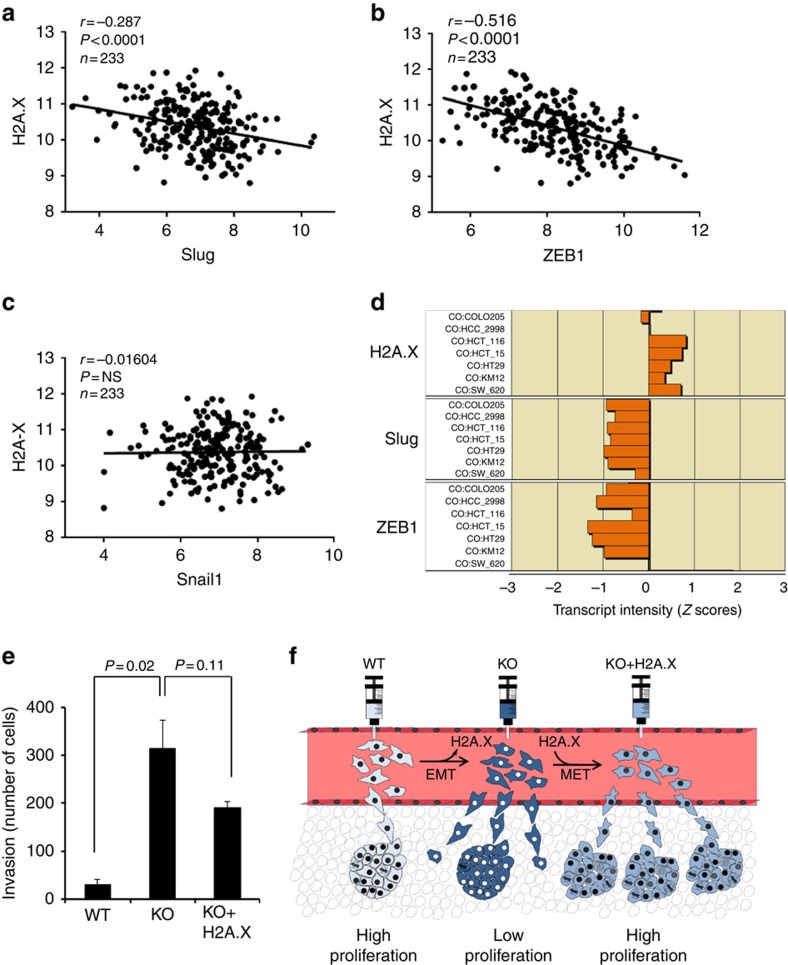
H2A.X expression is correlated with that of the EMT-inducing transcription factors Slug and ZEB1 in human adenocarcinoma. (**a**–**c**) Statistically significant correlation between transcript levels for H2A.X and Slug (**a**) or ZEB1 (**b**) in 233 adenocarcinoma samples in the TCGA COAD data set. No significant correlation was found between the level of H2A.X and SNAI1 (**c**) in the same data set. The *P* values were calculated using the non-parametric Mann–Whitney *t*-test. The Log 2 expression level is shown for the indicated genes. (**d**) Inverse correlation between the transcript levels of H2A.X (top) and Slug (middle) or ZEB1 (bottom) in the colon cancer subpanel of the NCI60. The *Z* score represents the transcript expression in standard deviation units relative to the gene-specific mean expression level. (**e**) Invasion rate in parental cells (WT), H2A.X knockout cells (KO) and revertant cells (KO+H2A.X). Data are means±s.d.; *n*=3. Statistical significance was determined by a two-tailed, unpaired Student's *t*-test. (**f**) Hypothetical model for the role of H2A.X in EMT and metastatic colonization.

Colorectal cancer (CRC) is the second- and third-most common cancer in women and men, respectively worldwide^49^,^50^. To investigate whether the role of H2A.X in the regulation of EMT-inducing transcription factors may provide clues for any clinical correlation, we conducted an expression level comparison of H2A.X, Slug and ZEB1 in human colon adenocarcinoma samples available in the *Cancer Genome Atlas* (*TCGA*) data sets. The data indicate that the transcript levels of H2A.X, Slug and ZEB1 are significantly correlated across all the colorectal carcinoma samples, based on the Pearson correlation coefficient (H2A.X and ZEB1, *r*=−0.516, *P*<0.0001; H2A.X and Slug, *r*=−0.287, *P*<0.0001, using the non-parametric Mann–Whitney *t*-test). No correlation was found with Snail1, an EMT-transcription factor found to be near the cut-off value of the fold-change level (−1.52506) in the microarray data sets (Fig. 6a–c, Supplementary Fig. 13). Furthermore, utilizing the NCI60 genome-wide expression data set (http://discover.nci.nih.gov/cellminer/), we found a strong negative correlation between H2A.X and Slug/ZEB1 expression in four of the seven colon cancer cell lines (Fig. 6d). Collectively, these observations indicate that Slug and ZEB1 may be selectively regulated by H2A.X in colon cancer, and further suggest that H2A.X may regulate EMT-inducing transcription factors during colon cancer progression.

From the physiological standpoint, EMT is a process that involves regulation by a set of factors ranging from alterations in cellular metabolism to contextual microenvironmental signals including inflammatory cytokines and oncogenic stressors among others^51^,^52^,^53^. Transforming growth factor beta (TGF-β) is one of these factors^54^,^55^. Genes profiles from Gene Expression Omnibus, GEO (*GSE17708*; ref. 56), indicate that the H2A.X gene is downregulated in the A549 lung adenocarcinoma cell line in a model of TGF-β-induced EMT. Hence, it could be anticipated that changes in the control of TGF-β regulation may be essential in maintaining H2A.X levels *in vivo*; and this provides a clue for the physiological conditions that may link H2A.X to EMT. On the other hand, additional hits including changes in H2A.X gene regulation during the cell cycle may also be critical for the role of H2A.X in EMT *in vivo*. For instance, it is known that H2A.X is synthesized in replication-dependent manner to ensure the presence of sufficient H2AX molecules in the replicating genome for efficient DSB detection^16^,^57^. Changes in the H2A.X levels may then occur during the cell cycle progression, and this raises the possibility that such variations could subsequently affect the EMT programme. Nevertheless, further investigation is needed to delineate well-defined physiological environments that may underlie the link between H2A.X level and the regulation of EMT genes *in vivo*.

In summary, our study provides novel evidence that H2A.X loss activates the EMT programme in two human colon cancer cell lines, making them more prone to invasion. However, H2A.X-deficient cells failed to exhibit enhanced metastatic colonization potential *in vivo*, perhaps because they have reduced proliferative ability, and are vulnerable to genotoxic stress. Restoration of H2A.X promotes partial EMT reversal, endowing cells with enhanced metastatic colonization ability, by enhancing their proliferative capacity and damage resilience, while maintaining their invasive properties (Fig. 6e,f). This may explain, in part, the lack of difference in H2A.X expression seen between the primary tumours and the matched liver metastases in human samples.

## Methods

### Cell culture and plasmid transient transfection

The human colorectal carcinoma cell lines HCT116 and HCT15 (parental lines obtained from ATCC and kept at the Frederick National Laboratory for Cancer Research, DTP, DCTD, NCI Tumor Repository), were grown at 37 °C with 5% CO_2_ in DMEM (Invitrogen, Carlsbad, CA, USA), supplemented with 10% fetal bovine serum (FBS, Atlanta Biologicals, GA, USA). The cells were used as models to study the changes associated with epithelial–mesenchymal transition. In addition, the cells were authenticated using short tandem repeat Identifiler, and tested for mycoplasma contamination. All media were supplemented with 2 mM glutamine, penicillin and streptomycin (Invitrogen). ATM inhibition was performed by incubating cells with KU-55933 (Tocris Biosciences, USA) for 72 h. For H2AX overexpression experiments, HCT116 parental cells were transfected using Lipofectamine 2000 (Thermo Scientific, Hudson, NH, USA) following the manufacturer's instructions. pCMV6-ENTRY and pCMV6-H2AX-Myc-DDK vectors were purchased from Origene (Origen Technologies, Inc., Rockville, MD, USA).

### siRNA transfection

Small RNA interference (siRNA) transfection experiments were performed with Dharmafect (Thermo Scientific). A SMARTpool consisting of four short sequences of siRNA specific for Slug, ZEB1, H2A.X (H2AFX) or macroH2A.1 (H2AFY) (Dharmacon, Thermo Scientific) along with Scrambled control (nontargeting siRNA) were used under conditions specified by the manufacturer. siRNAs sequences are listed in the Supplementary Table 1.

### Generation of stable H2A.Z and H2A.X knocked-down cells

For lentiviral infection, HEK 293T cells were used for virus package according to the manufacturer's instructions. To obtain H2A.Z- or H2A.X-shRNA knockdown cells, or the control cell line, HCT116 parental cells infected with pLKO.1-puro-H2A.ZshRNA (shH2A.Z) or pLKO.1-puro-H2A.XshRNA (shH2A.X); or control vector were selected with puromycin (0.8 μg ml^−1^) for 3 weeks and the expression of H2A.X or H2A.Z were examined by western blotting. Briefly, the lentivirus packaging system was used as follows: lentiviral constructs that expressed shRNAs targeting H2A.Z/H2A.X; or scrambled shRNAs were co-transfected with lentiviral packaging plasmids psPax2 and pCMV-VSVG into HEK 293T cells by *LipoD293DNA in vitro* transfection reagent (SignaGen Laboratories, Gaithersburg, MD, USA) according to the manufacturer's instructions. At 48 h post transfection, the culture medium was collected to be incubated with HCT116 cells in the presence of polybrene (5 μg ml^−1^). At 48 h post infection, infected cells were either collected for gene and protein expression analysis or selected with puromycin to establish stable clones. ShH2A.Z was obtained from Sigma-Aldrich, Saint Louis, MO, USA and shH2A.X: #TRCN0000073281 (Open Biosystems/Dharmacon, Thermo Scientific) was a kind gift of Dr Judith Campisi, and has been previously tested for further validation^58^.

### Generation of cells expressing H2A.X-WT or H2A.X-S139A

For retroviral infection, HEK 293T were used for virus packaging according to the manufacturer's instructions. Briefly, retroviral constructs p-BABE-puro or pBABE-puro-H2A.X-WT or pBABE-puro-H2A.X-S139A, pVPack-VSVG and pVPack-GP were transfected into HEK 293T cells using Lipofectamine 2000 according to the manufacturer's instructions; viral particles were harvested at 48 h post transfection. The cells were infected with virus for 48 h in the presence of DEAE-dextran (10 μg ml^−1^). The infected cells were either collected for gene and protein expression analysis or selected to establish stable clones. For the plasmid pBABE-puro-H2A.X construct, H2A.X was amplified between BamHI and EcoRI sites from pCR2.1 vector using the following primers: forward primer: 5′-GTCGGATCCATGTCGGGCCGCGG-3′ and reverse primer: 5′-GTAGAATTCTTAGTACTCCTGGGAGGCCTGG-3′. The PCR product was digested and subcloned into p-BABE-puro vector. S139A point mutation in H2A.X was introduced using the QuikChange mutagenesis kit by Genewiz, Inc.

### ChIP analyses

HCT116 cells (shCTRL, shH2A.X, parental and H2A.X KO) were processed for ChIP using Magna ChIP A/G kit according to the manufacturer's instructions (Millipore, MA, USA). Briefly, 15 × 10^6^ (ref. 6) cells were treated with formaldehyde to crosslink chromatin, lysed using buffer provided in the kit. Sheared DNA corresponding to 1 million cells was incubated overnight at 4 °C with primary antibodies for H2A.X (ab20669, Abcam, Cambridge, MA, USA), or H3K9ac (ab4441, Abcam), or H3K27ac (ab4729, Abcam) or normal rabbit IgG (sc-2027, Santa Cruz Biotechnology, Santa Cruz, CA, USA). The chromatin was then washed, the crosslinking was reversed and the DNA was purified using phenol–chloroform precipitation before PCR analysis. The following sets of primers mapping the promoters regions were used: Slug Forward 5′-TCCGAACAAACCCTCACATAG-3′; Slug reverse 5′-CACACAAACTGGAACCTGGA-3′; ZEB1 Forward 5′-GGCGCAATAACGGTGAGT-3′; ZEB1 reverse 5′-ACTTTCCCACTCCACTTTGC-3′; GAPDH forward 5-TACTAGCGGTTTTACGGGCG-3′; GAPDH reverse 5′-GAGGCTGCGGGCTCAATTT-3′.

### Luciferase assay

The promoter luciferase reporter assay was performed as previously described^59^. The cells were seeded in triplicate into a 96-well plate and cultured for 24 h. The LightSwitch Promoter Reporter plasmids SNAI2, ZEB1, GAPDH and control plasmid ACTB (Active motif, Carlsbad, CA, USA); were transfected into the cells using Lipofectamine 2000 reagent (Life Technologies; Grand Island, NY, USA). Luciferase activity was measured 24 h after transfection using the LightSwitch Luciferase Assay System (Switchgear Genomics), following the manufacturer's instructions.

### Transwell migration and invasion assays

Matrigel inserts were hydrated for 2 h in 500 μl serum-free DMEM; then suspensions of 8 × 10^4^ cells were seeded in triplicate into each well of the 24-well plate format BD BioCoat Control and Matrigel Invasion Chambers (BD Biosciences, San Jose, CA, USA) and incubated for 48 h at 37 °C in 5% CO_2_. Serum-enriched media was used as chemoattractant. Membranes were subsequently fixed with methanol, stained with crystal violet and cells present on the underside of the membrane were counted^60^.

### Real-time PCR

Total RNA was extracted from cells using RNeasy Mini Kit (Qiagen, Valencia, CA, USA) according to the manufacturer's instructions. Quality of RNA preparation, based on the 28S/18S ribosomal RNAs ratio, was assessed using the RNA 6000 Nano Lab-On-chip (Agilent Technologies, Palo Alto, CA, USA). Reverse transcription and Real-time PCR (RT-PCR) were performed as previously described^61^. Oligonucleotides were pre-designed, validated and considered to be proprietary information by Thermo Fisher Scientific. However, the assays IDs are available and are referenced as follows: ZEB1 (Hs00232783_m1), ITGB4 (Hs00173995_m1), Slug (Hs00161904_m1), TGFB2 (Hs00234244_m1), THBS1 (Hs00962908_m1), VCAN (Hs00171642_m1), CDH1 (Hs01023894_m1), GAPDH (Hs02758991_g1), HPRT1 (Hs02800695_m1), RPLP0 (Hs99999902_m1), 18S (Hs03928985_g1) and VIM (Hs00958111_m1).

### Clonogenic assay

Cell survival was assessed by colony formation assay as previously described^62^. The cells were trypsinized and identical numbers of HCT116 parental cells (WT), H2A.X knockout cells (KO) and H2A.X revertant cells (KO+H2A.X) were plated on 35 mm dishes. Six hours after seeding, the cells were irradiated with doses ranging from 1 to 4 Gy in a Mark-1 γ-irradiator (JL Shepherd and Associates, San Fernando, CA, USA) at a dose rate of 2.2 Gy min^−1^. After 15 days of incubation, the colonies were fixed with methanol for 10 min and then stained with Coomassie blue. Colonies with >50 cells were counted under a dissection microscope. Clonogenic survival curves were constructed from at least three independent experiments.

### Proliferation assay

Two-millilitres of 2.5 × 10^4^ cells were seeded in three replicates into each well of six-well cell culture plates (Corning, Inc.). The cells were collected following the time point for counting.

### Microarray

Total RNAs from HCT116 cells infected with scrambled shRNAs (shCTRL) or with shRNAs targeting H2A.X (shH2A.X) were extracted using RNeasy Mini Kit (Qiagen) following the manufacturer's instructions. All the RNAs were QC-tested and used at LMT/Affymetrix Group, NCI-Frederick, MD, USA, to perform Affymetrix GeneChip Human Gene 2.0 ST array (*n*=5 for each group). Affymetrix Expression Console was used to generate CHP files from CEL files. Then, the CEL files were loaded into Partek Genomics Suite software, version 6.6 for normalization and differential expression analysis. The NCBI/GEO accession number is GSE75444; and data are available at http://www.ncbi.nlm.nih.gov/geo/query/acc.cgi?token=mdcbmqikhdcvtsp&acc=GSE75444. Biological interpretation and pathway analysis was performed with the IPA tool.

### Alkaline comet assay for analysis of DNA damage

The cell samples (HCT116 parental cells, H2A.X knockout cells and H2A.X revertant cells) are handled under dimmed or yellow light to prevent DNA damage from ultraviolet light. Briefly, the cells were collected with trypsin, washed once with PBS and resuspended in 1 × TE. The cells were then mixed with 37 °C prewarmed/melted 1.6% low-melting agarose to make a final agarose concentration of 0.85%. The mixture was spread over the sample area on FLARE slides (Trevigen, Gaithersburg, MD, USA). After lysis, the slides were transferred into alkali solution (pH >13) for 1 h at room temperature in the dark, then electrophoresis was performed (0.7 V cm^−1^ per 300 mA) for 1 h at room temperature in the dark. After electrophoresis, the slides were washed three times for a total of 20 min with water. Then, the slides were washed in chilled 70% alcohol for 15 min. Each slide was stained with 50 μl of 1:10,000 diluted SyBr green solution (Trevigen, Gaithersburg MD); and then air-dried overnight in the dark. The individual cells with or without comet were visualized using an Olympus fluorescence microscope. The analysis was done by scoring at least 50 comets per sample using the CometScore software package (Tritek Corp., Sumerduck VA, USA). The results are shown as means±s.e.

### Immunofluorescence

The cells were fixed for 20 min with freshly prepared 2% paraformaldehyde in phosphate-buffered saline (PBS). After washing with PBS, the cells were permeabilized with pre-chilled ethanol 70% for 20 min. The samples were then incubated for 30 min with 5% bovine serum abumin (BSA) in PBS containing 0.5% Tween-20 and 0.1% Triton X-100 (PBS-TT) for blocking. The cells were incubated for 2 h at room temperature with primary antibodies diluted in 1% BSA–PBS–TT: γ-H2A.X (1:500; 05-636, Millipore); 53BP1 (1:500; NB100-305, Littleton, CO, USA); E-cadherin (1:250; ab76055, Abcam); H2A.X (1:500; ab20669, Abcam); ZEB1 (1:100; sc-25388, Santa Cruz Biotechnology); Slug (1:100; sc-15391, Santa Cruz Biotechnology); and β-catenin (1:500; 610153, BD Biosciences). After washing with PBS–TT, the cells were stained with goat anti-rabbit Alexa fluor488 or goat anti mouse Alexa fluor555 diluted in 1% BSA–PBS–TT for 1 h at room temperature. Finally, the cells were washed in PBS and coverslips were mounted for analysis. Fluorescent images were captured using a confocal microscope (Nikon PCM2000).

### Western blots

The cells were washed twice with PBS, directly solubilized in denaturing sample buffer and then subjected to SDS polyacrylamide gel electrophoresis. Proteins were electrotransferred to 0.2 μm Protan BA 83 nitrocellulose sheets (Invitrogen) for immunodetection with the following primary antibodies: H2A.X (1:2,000; ab20669, Abcam);H2A.X (1:2,000; NB100-638, Littleton, CO, USA); β-catenin (1:2,500; 610153, BD Biosciences); E-cadherin (1:2,500; ab76055, Abcam); ZEB1 (1:500; sc-25388, Santa Cruz Biotechnology); Slug (1:500; 9585S, Cell Signaling Technology Inc.); Integrin-β4 (1:2,000; sc-9090, Santa Cruz Biotechnology); macroH2A.1 (1:1,000; 07-219, Millipore); H2A.Z (1:2,000; ab150402, Abcam); Tubulin (1:2,000; ab6046, Abcam); c-Myc (1;2000; M4439, Sigma-Aldrich) and actin (1:2,000; A5060, Sigma-Aldrich). Immune complexes were detected with horseradish peroxidise-coupled anti-rabbit or anti-mouse IgG antibodies (Amersham, GE Healthcare, Pittsburgh, PA, USA). The cropped images for the western blots and molecular weight are shown in the main and supplementary figures; however, the uncropped scan for each blot is shown in the Supplementary Fig. 14).

### Gene deletion using CRISPR/Cas9 system

H2A.X gene (H2AFX) deletion was performed using CRISPR/Cas9 system as previously described^63^,^64^,^65^. Briefly, the H2AFX-specific guide RNA sequence was chosen from http://crispr.mit.edu database; and inserted into the pX330 vector (Addgene, Cambridge, MA, USA). For the donor plasmid, the left and the right homology arms were PCR-amplified from human primary fibroblasts genomic DNA. Both the arms along with antibiotic resistance ORF (hygromycin) were inserted between BamHI and EcoRI sites of the PCR2.1 vector (Life Techonologies; Grand Island, NY, USA).

### Experimental lung metastasis model

All animal procedures were performed according to protocols approved by NCI-Frederick Animal Care and Use Committee (ACUC). Tail vein injection was performed as previously described^66^,^67^. Four to six-week-old male athymic nude mice (Charles River Laboratories, Frederick, MD, USA) were injected with HCT116 parental cells (WT), H2A.X knockout cells (KO) and H2A.X revertant cells (KO+H2A.X) via the lateral tail vein using 25-gauge needles and followed up for lung metastases burden. In brief, 5 × 10^6^ cells suspended in 500 μl PBS were injected into the tail vein of each mouse. After 4 weeks, the animals were killed and examined macroscopically and microscopically for the presence of metastases.

### Statistical analysis

Statistical analyses were performed using GraphPad Prism 6 software (GraphPad Software) and Microsoft Excel 2010. Parametric data were analysed using a two-tailed *t*-test. A value of *P*<0.05 was considered statistically significant. Data are presented as mean±s.d.

## Additional information

**Accession codes**: Microarray data have been deposited in the NCBI/GEO database under the accession code GSE75444.

**How to cite this article**: Weyemi, U. *et al.* The histone variant H2A.X is a regulator of epithelial–mesenchymal transition. *Nat. Commun.* 7:10711 doi: 10.1038/ncomms10711 (2016).

## Supplementary Material

Supplementary Information Supplementary Figures 1-14 and Supplementary Table 1
