# Supplementary material for: The histone variant H2A.X is a regulator of the epithelial–mesenchymal transition
Source: Nat Commun. 2016 Feb 15;7:10711. doi: 10.1038/ncomms10711 (PMC4756313; doi:10.1038/ncomms10711)
Supplement: Supplementary Information — Supplementary Figures 1-14 and Supplementary Table 1 [file ncomms10711-s1.pdf]

Supplementary information

The histone variant H2A.X is a regulator of Epithelial–Mesenchymal Transition

Urbain Weyemi, Christophe E. Redon, Rohini Choudhuri, Towqir Aziz, Daisuke Maeda, Myriem Boufraqech, Palak R. Parekh, Taresh K. Sethi, Manjula Kasoji, Natalie Abrams, Anand Merchant, Vinodh N. Rajapakse, and William M. Bonner.

Correspondence should be addressed to UW (weyemiurbain@yahoo.fr) or WMB (bonnerw@mail.nih.gov).

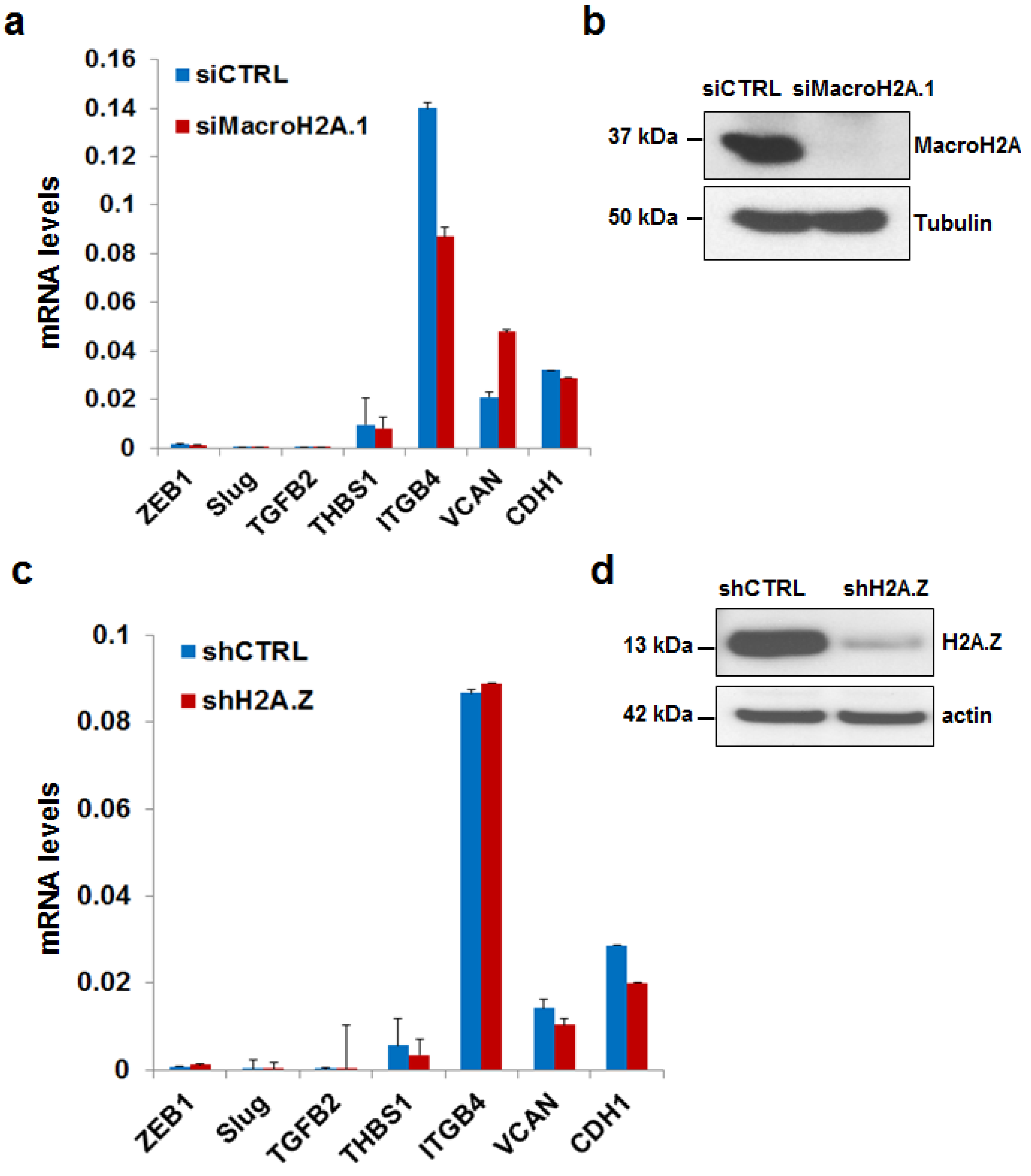

Supplementary figure 1: MacroH2A.1 or H2A.Z silencing does not affect EMT markers. (**a**) Parental HCT116 cells were transfected for 5 days with siRNA control (siCTRL) or with siRNA targeting histone variant macroH2A.1. Transcript levels of several EMT markers were analyzed by real time PCR (RT-PCR). (**b**) Western blot analysis reveals an efficient knockdown of macroH2A.1 protein level. (**c, d**) The same experiments were performed using HCT116 parental cells expressing scrambled shRNA or shRNA targeting the histone variant H2A.Z. Expression values are relative fold change for gene

transcripts of ZEB1, Slug, VIM, ITGB4, VCAN and CDH1, normalized to 18S RNA (gene/18S ratio). Error bars represent the S.D. ($n = 3$).

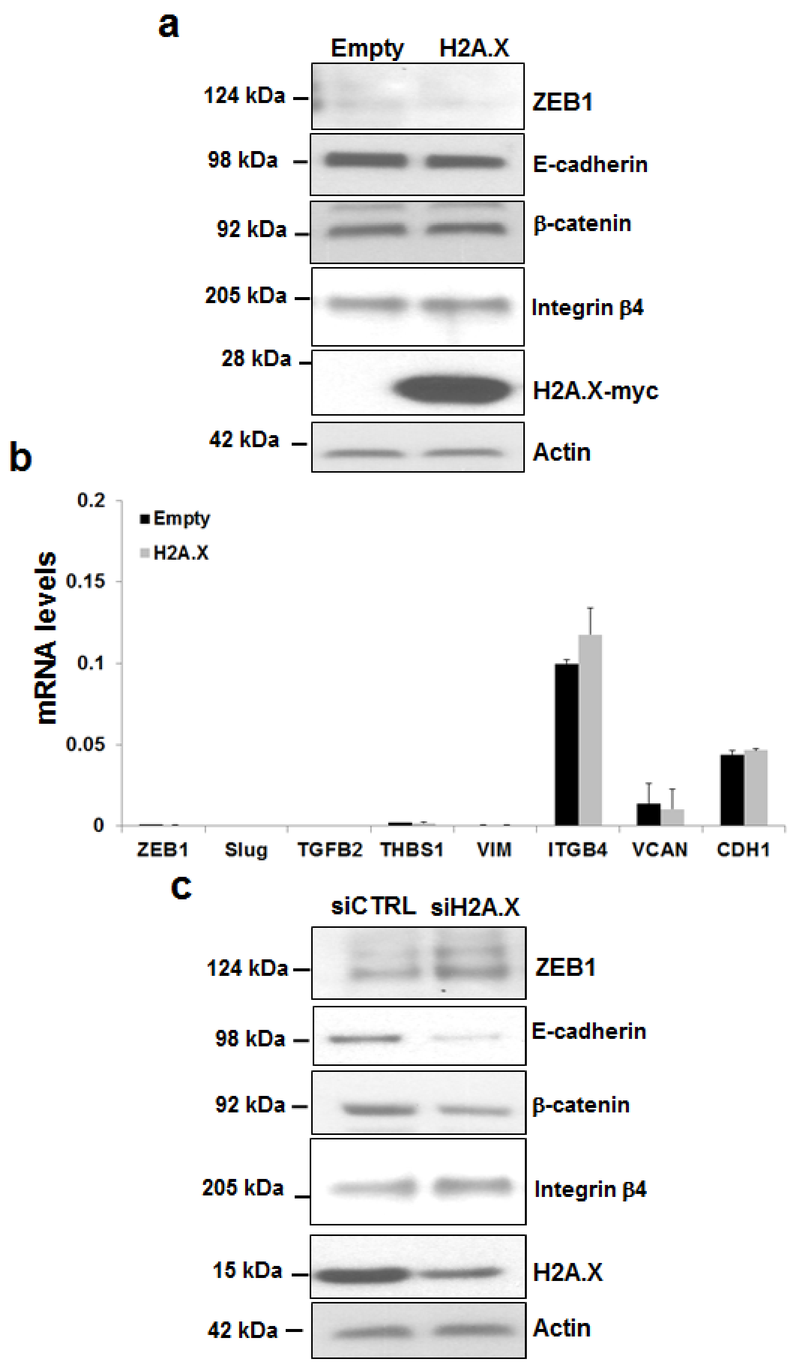

Supplementary figure 2: overexpression of H2A.X does not affect EMT markers. (**a**) HCT116 parental cells were transfected with empty vector (pCMV6-Entry) or with Myc-DDK-tagged-Human H2A.X vector for 5 days. ZEB1, E-cadherin, β-catenin, ITGB4 (integrin β4) expression was analyzed by

western blotting. Overexpressed H2A.X protein was detected using c-myc antibody and actin was used as loading control. (**b**) Transcript levels of cells in (a) were analyzed by real time PCR. Expression values are relative fold change for gene transcripts of ZEB1, Slug, TGFB2, THBS1, VIM, ITGB4, VCAN and CDH1, normalized to GAPDH, RPLP0 and HPRT1. Error bars represent the S.D. (n = 3). (**c**) HCT116 parental cells were transfected with scrambled siRNA (siCTRL) or with siRNA targeting H2A.X (siH2A.X) as in overexpression experiments (5 days). H2A.X, ZEB1, E-cadherin, β-catenin, integrin β4 expression was analyzed by western blotting and actin was used as loading control.

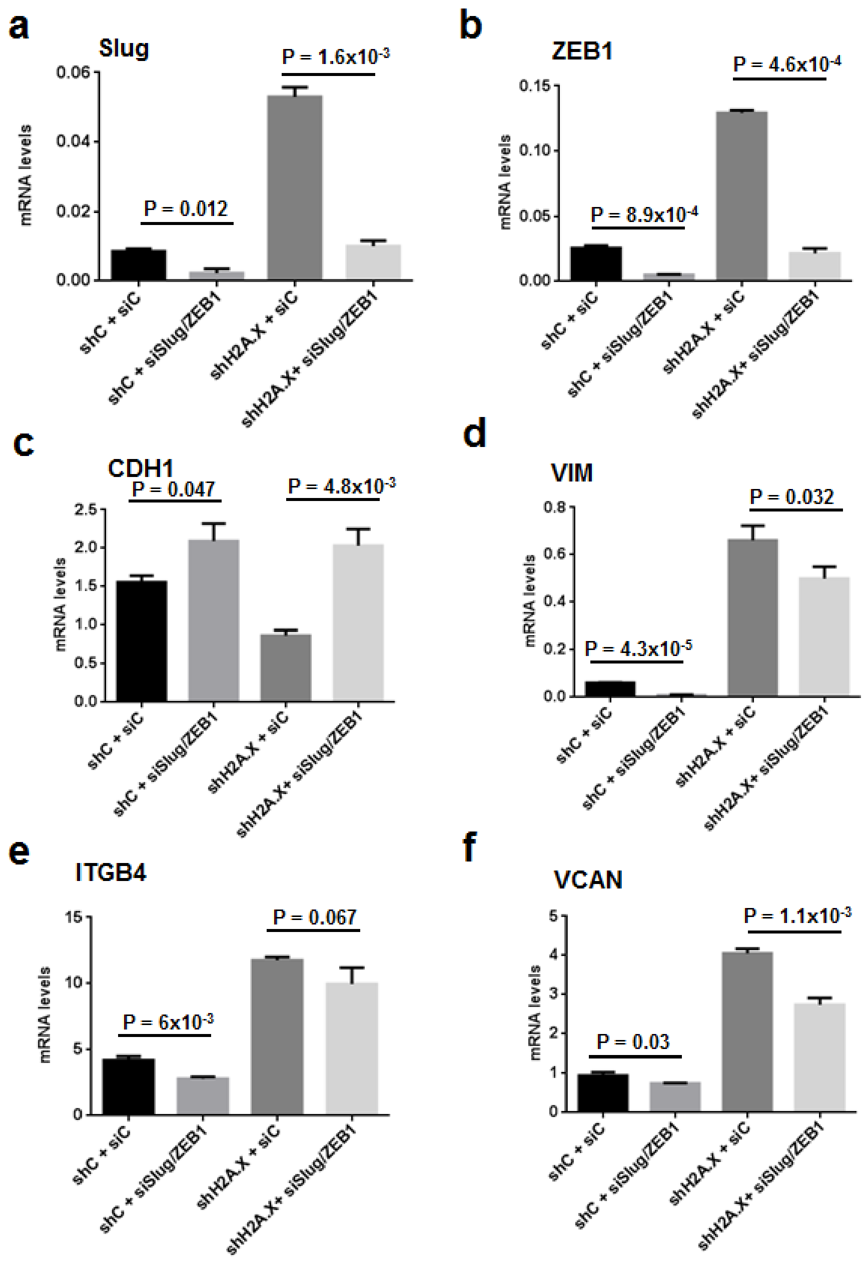

Supplementary figure 3: The co-silencing of Slug and ZEB1 represses EMT. HCT116 control cells (shC) and cells deficient for H2A.X (shH2A.X) were transfected with scrambled siRNA (siC) or with a pool of siRNAs targeting Slug and ZEB1 (siS/Z) for 3 days. Transcript levels were analyzed by real time PCR. Expression values are relative fold change for gene transcripts of Slug (**a**), ZEB1 (**b**), CDH1 (**c**), VIM (**d**), ITGB4 (**e**) and VCAN (**f**), normalized to 18S RNA (gene/18S ratio). Error bars indicate the S.D. (n = 3). Statistical significance was determined by a two-tailed, unpaired Student's t-test.

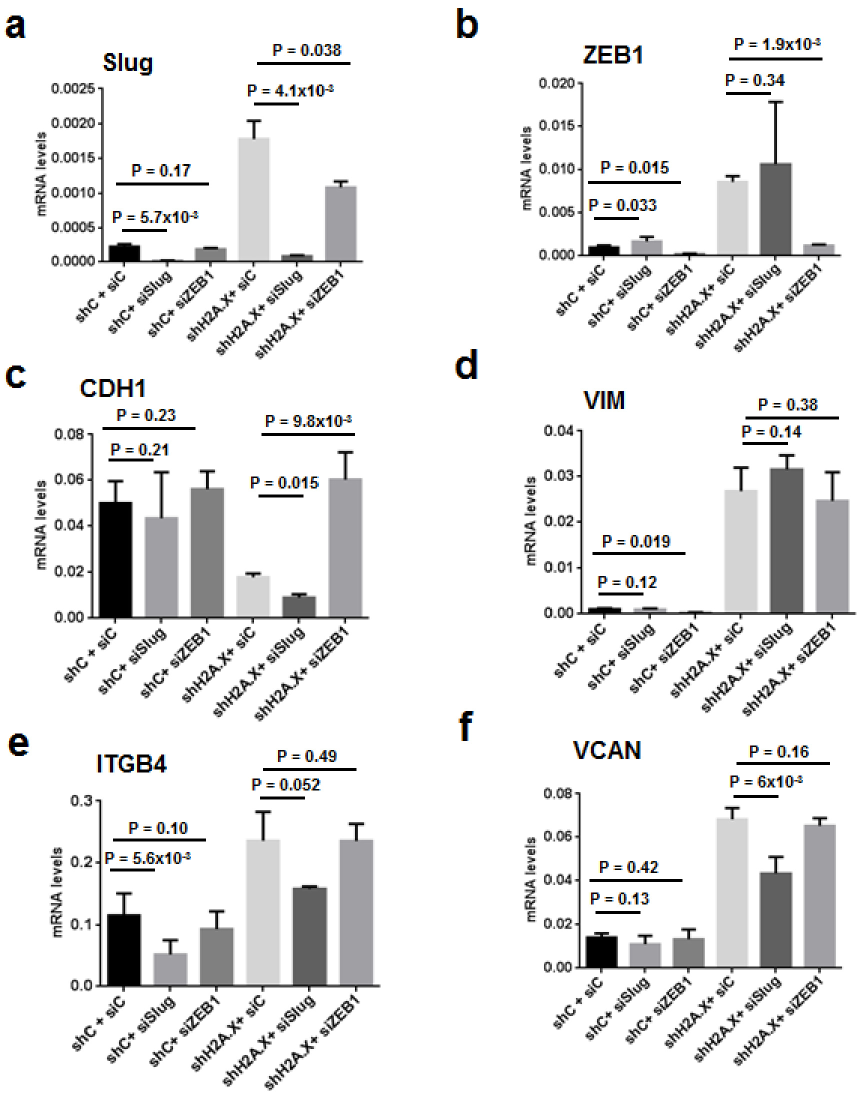

Supplementary figure 4: Effects of Slug or ZEB1 silencing on EMT markers. HCT116 control cells (shC) and cells deficient for H2A.X (shH2A.X) were transfected with scrambled siRNA (siC) or with siRNA targeting Slug (siSlug) or ZEB1 (siZEB1) for 3 days. Transcript levels were analyzed by real time PCR. Expression values are relative fold change for gene transcripts of Slug (**a**), ZEB1 (**b**), CDH1 (**c**), VIM (**d**), ITGB4 (**e**), and VCAN (**f**). GAPDH, RPLP0 and HPRT1 were used as housekeeping genes.

Error bars indicate the S.D. (n = 3). Statistical significance was determined by a two-tailed, unpaired Student's t-test.

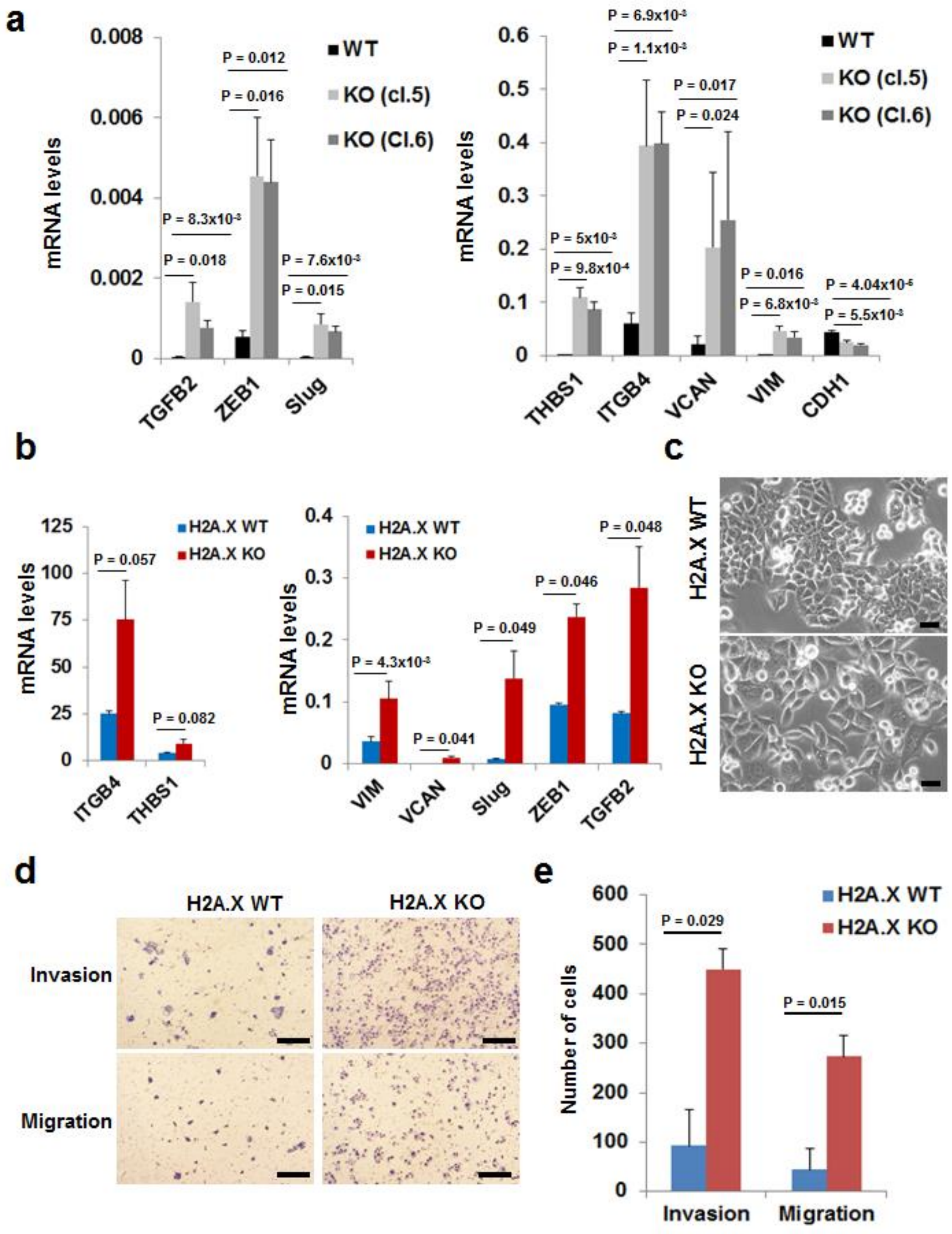

Supplementary figure 5: Analysis of EMT markers in HCT116 and HCT15 colon cell lines. (**a**) HCT116 parental cells (WT) and H2A.X knockout (KO) clones were cultured for 3 days and RNA was used for transcript levels analysis by real time PCR. Expression values are relative fold change for gene transcripts normalized to GAPDH, RPLP0 and HPRT1. Cl.5, clone #5 and cl.6, clone #6. Error bars indicate the S.D. (n=3). Statistical significance was determined by a two-tailed, unpaired Student's *t*-test. (**b**) Increased expression of EMT markers in HCT15 H2AX KO cells. HCT15 parental cells (H2A.X WT) and H2A.X knockout (H2A.X KO) cells were cultured as in (a), and RNA was used for transcript levels analysis by real time PCR. Expression values are relative fold change for gene transcripts normalized to GAPDH, RPLP0 and HPRT1. Error bars indicate the S.D. (n=3). Statistical significance was determined by a two-tailed, unpaired Student's *t*-test. (**c**) Photomicrographs of HCT15 parental cells (H2A.X WT) and H2A.X knockout (H2A.X KO) cells, Scale bars, 20 μm. (**d, e**) HCT15 H2A.X knockout cells exhibit increased invasion and migration ability in transwell invasion/migration assays (**d**, photomicrograph, Scale bars, 100 μm; **e**, quantitation, Error bars indicate the S.D. (n=3). Statistical significance was determined by a two-tailed, unpaired Student's *t*-test.

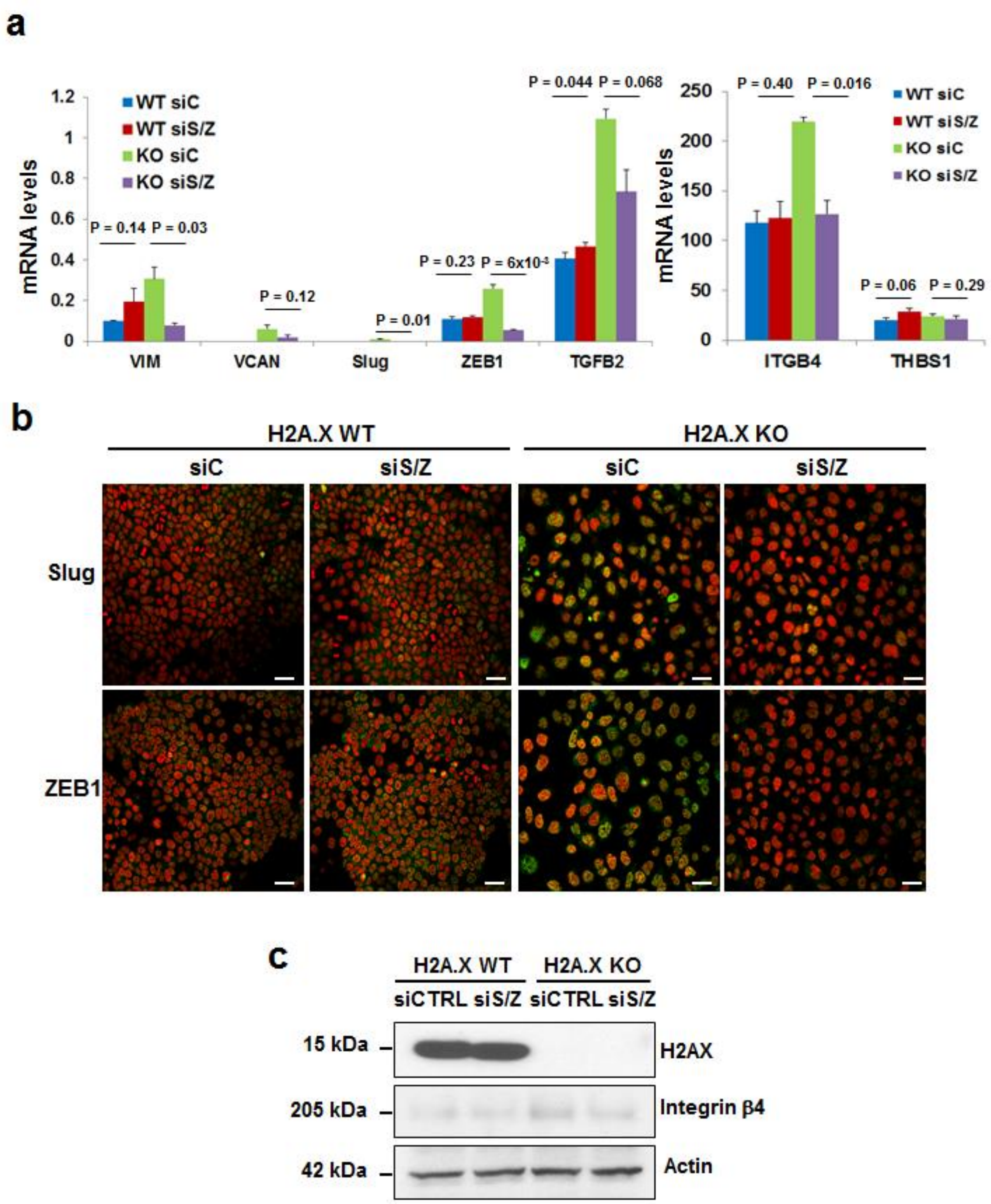

Supplementary figure 6: The co-silencing of Slug and ZEB1 represses mesenchymal genes in HCT15 cells. (**a**) Illustration of mesenchymal markers repression (VIM, TGFB2, ITGB4) by the co-silencing of Slug and ZEB1 in HCT15 H2A.X KO cells. HCT15 parental cells (WT) and H2A.X knockout cells (KO) were transfected with scrambled siRNA (siC) or with a pool of siRNAs targeting both Slug and ZEB1 (siS/Z) for 3 days. The transcript levels were analyzed by real time PCR. Expression

values are relative fold change for gene transcripts normalized to the average levels of GAPDH and HPRT1 genes. Error bars indicate the S.D. (n=3). Statistical significance was determined by a two-tailed, unpaired Student's *t*-test. (**b**) Staining of Slug and ZEB1 by immunofluorescence in HCT15 parental cells (H2A.X WT) and H2A.X knockout cells (H2A.X KO) transfected for 3 days with control siRNA (siC) or a pool of siRNAs against SLUG and ZEB1 (siS/Z). Nuclei were counterstained with propidium iodide (red); Scale bars, 20 μm. (**c**) Validation of H2A.X depletion in the cells used in (a) and (b) by western blotting. Actin was used as loading control and integrin β4 was analyzed as a marker for mesenchymal trait.

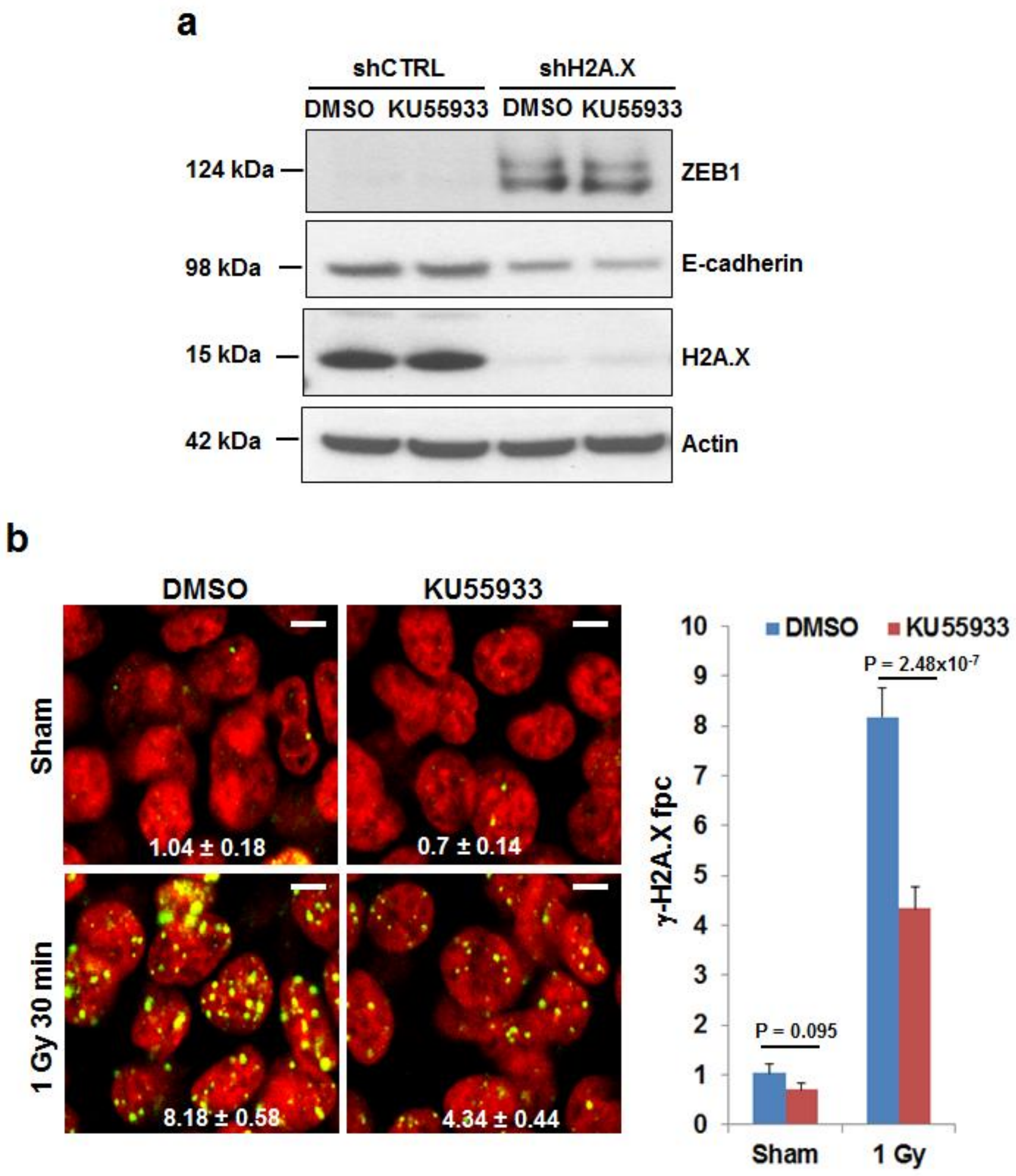

Supplementary figure 7: ATM inhibition does not decrease ZEB1 in H2A.X-deficient cells. (**a**) HCT116 control cells (shCTRL) and cells deficient for H2A.X (shH2A.X) were treated with DMSO or with KU55933 (10 µM) for 3 days and cells were used for protein expression analysis by western blot. (**b**) KU55933 efficacy was monitored by the detection of γ-H2AX foci number per cell (fpc) by immunofluorescence. Nuclei are counterstained with propidium iodide (red). Left panel, Representative

images, Scale bars, 20 μm; right panel, Quantification. . Error bars indicate the S.E.M. (n = 50). Statistical significance was determined by a two-tailed, unpaired Student's t-test.

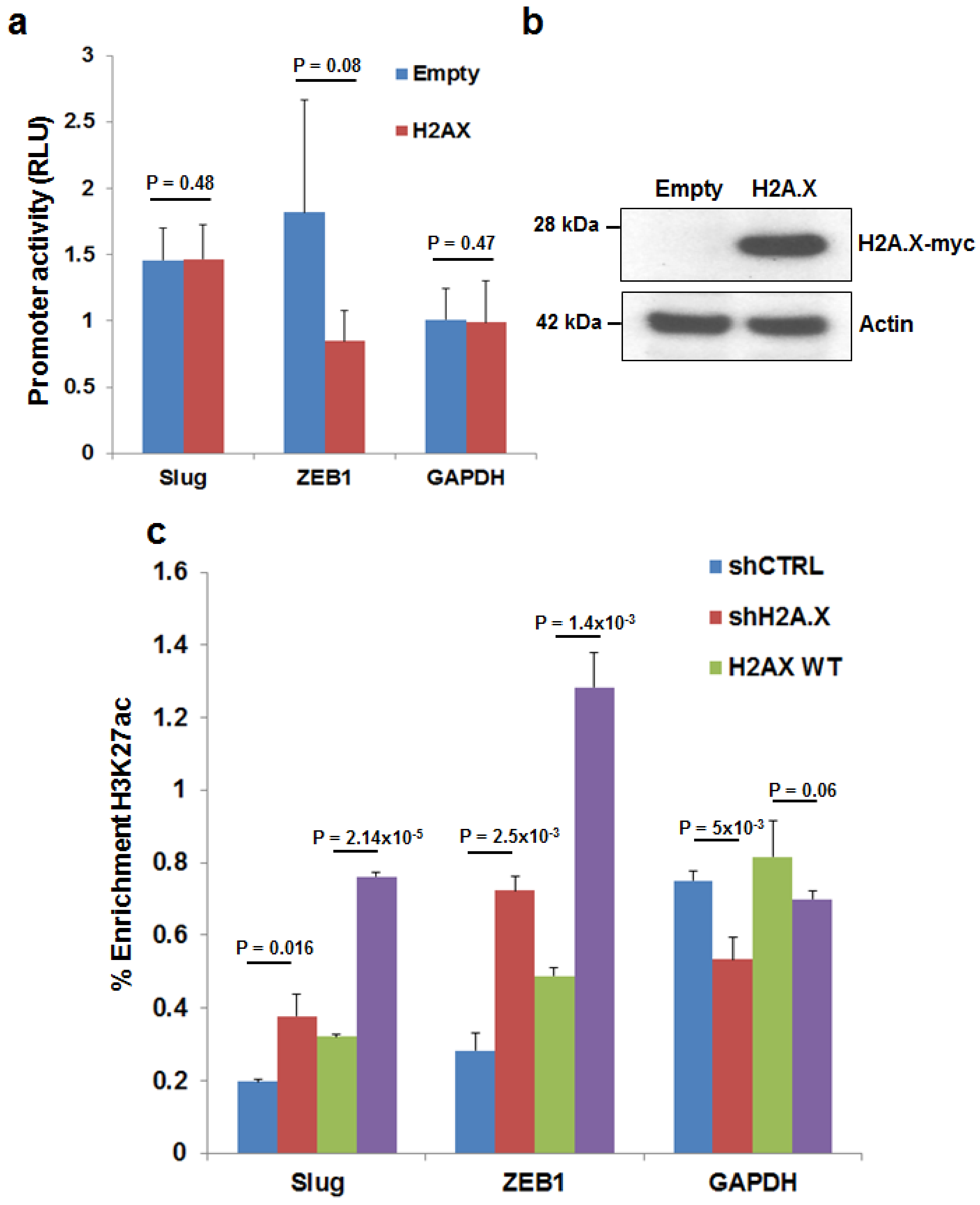

Supplementary Figure 8: Effects of H2A.X on Slug and ZEB1 transcriptional regulation.. (a) HCT116 parental cells were transfected with empty vector (pCMV6-Entry) or with Myc-DDK-tagged-

Human H2A.X vector (H2A.X) for 4 days. Slug, ZEB1 and GAPDH promoter activities were accessed by luciferase reporter assay. Error bars represent the S.E.M (n = 3). (n.s.) non-significant. Statistical significance was determined by a two-tailed, unpaired Student's *t*-test. (**b**) H2A.X expression was analyzed by western blotting. Overexpressed H2A.X protein was detected using c-myc antibody and actin was used as loading control. (**c**) H2A.X deletion enhances the enrichment of H3K27ac (an active transcription mark) to Slug and ZEB1 promoters. Chromatin from control cells (shCTRL) and cells silenced for H2A.X (shH2A.X) or parental cells (H2A.X WT) and H2A.X knockout cells (H2A.X KO) was immunoprecipitated with anti-H3K27ac antibody. The purified DNA was analyzed by real time PCR using primers amplifying across Slug, ZEB1 and GAPDH promoters. Results are presented as percentage of total input DNA precipitated. GAPDH promoter serves as an internal control. Error bars represent the S.E.M. (n = 3). Statistical significance was determined by a two-tailed, unpaired Student's *t*-test.

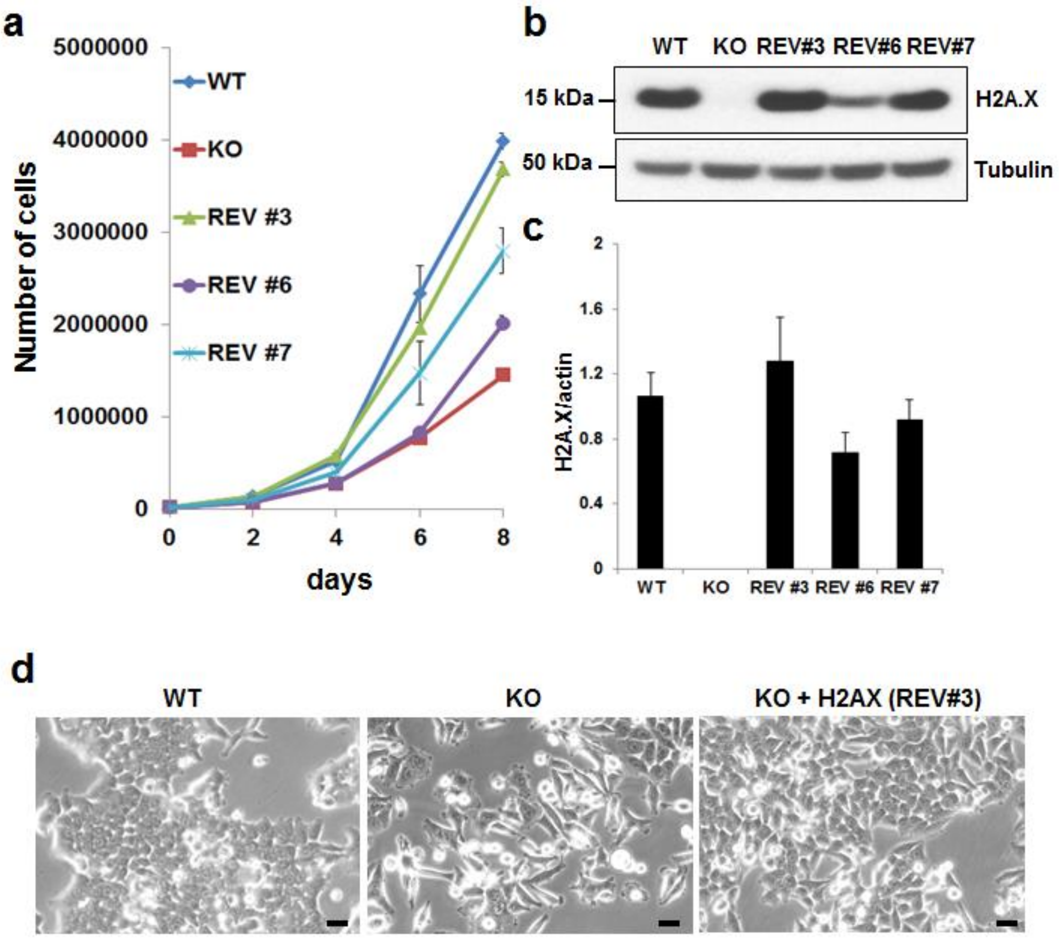

Supplementary figure 9: Effect of H2A.X restoration on proliferation and cell morphology (**a**) Quantification of the proliferation rate of HCT116 parental cells (WT), H2A.X knockout cells (KO) and 3 clones of revertant cells (REV#3, REV#6 and REV#7) with different H2A.X expression levels. Cells with lower H2A.X level (REV#6), exhibit defective proliferative ability; while cells with fully restored H2A.X display higher proliferative abilities (REV#3). REV#3 clone was used as revertant (KO + H2A.X) *in vitro* as well as *in vivo*, as its exhibits a similar proliferation rate as parental cells. . Error bars indicate the S.D. (*n* = 3) (**b, c**) H2A.X protein levels detected by western blotting for cells shown in (a). (**b)** Representative image; (**c)** quantification. (**d**) Photomicrographs of HCT116 parental cells (WT), H2A.X knockout cells (KO) and H2A.X knockout cells in which H2A.X expression was restored (KO + H2A.X). Parental cells display epithelial shape with tight cell-to-cell contact, while cells deficient for H2A.X are more scattered

with mesenchymal-like phenotype. Ectopic expression of H2A.X partly restores the epithelial phenotype; Scale bars, 20 μm.

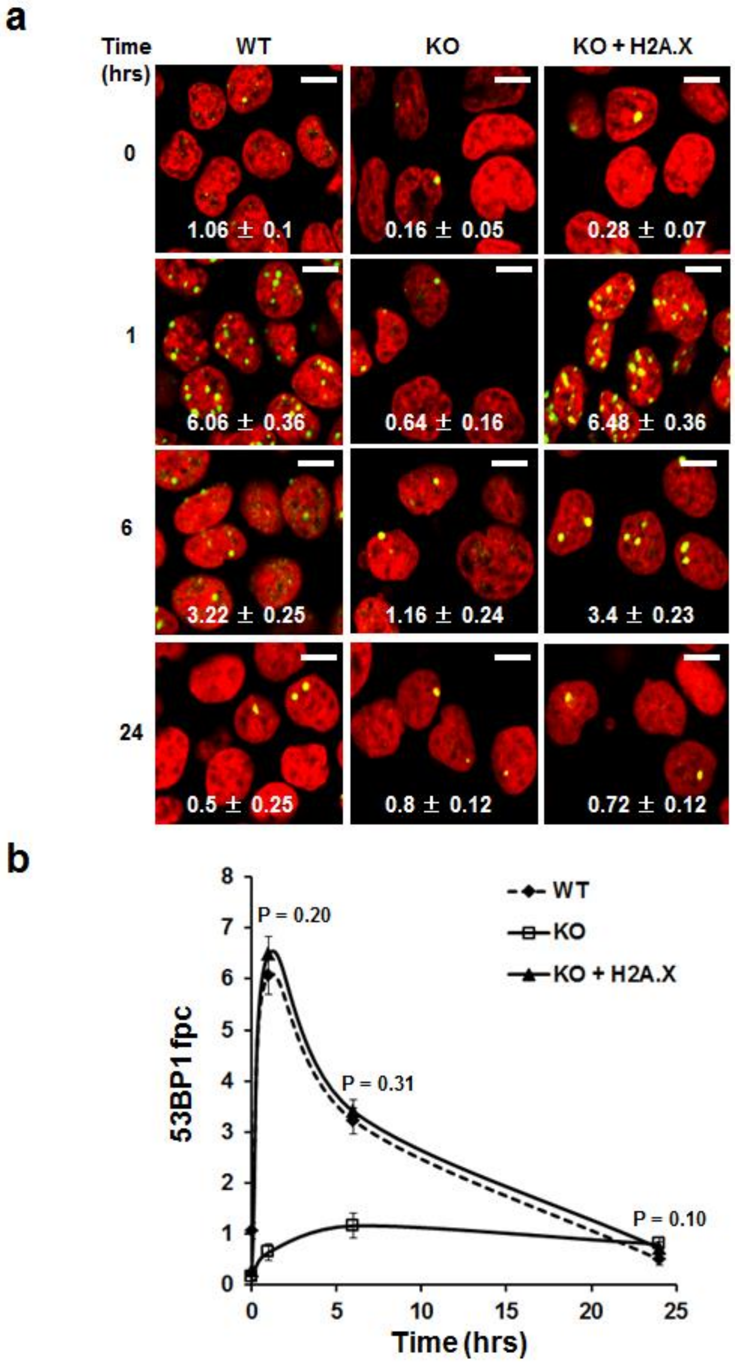

Supplementary figure 10: Ectopic expression of H2A.X reestablished efficient DNA repair process. HCT116 cell cultures (WT, KO and KO + H2A.X) were exposed to 1 Gy of ionizing radiation. DNA damage levels were analyzed by counting 53BP1 foci (green) in nuclei counterstained for DNA (red). (**a**) Representative images, Scale bars, 20 μm. (**b**) Quantitation of 53BP1 foci per cell. Error bars indicate the S.E.M. (n = 50). fpc. foci per cell.Statistical significance was determined by a two-tailed, unpaired Student's t-test.

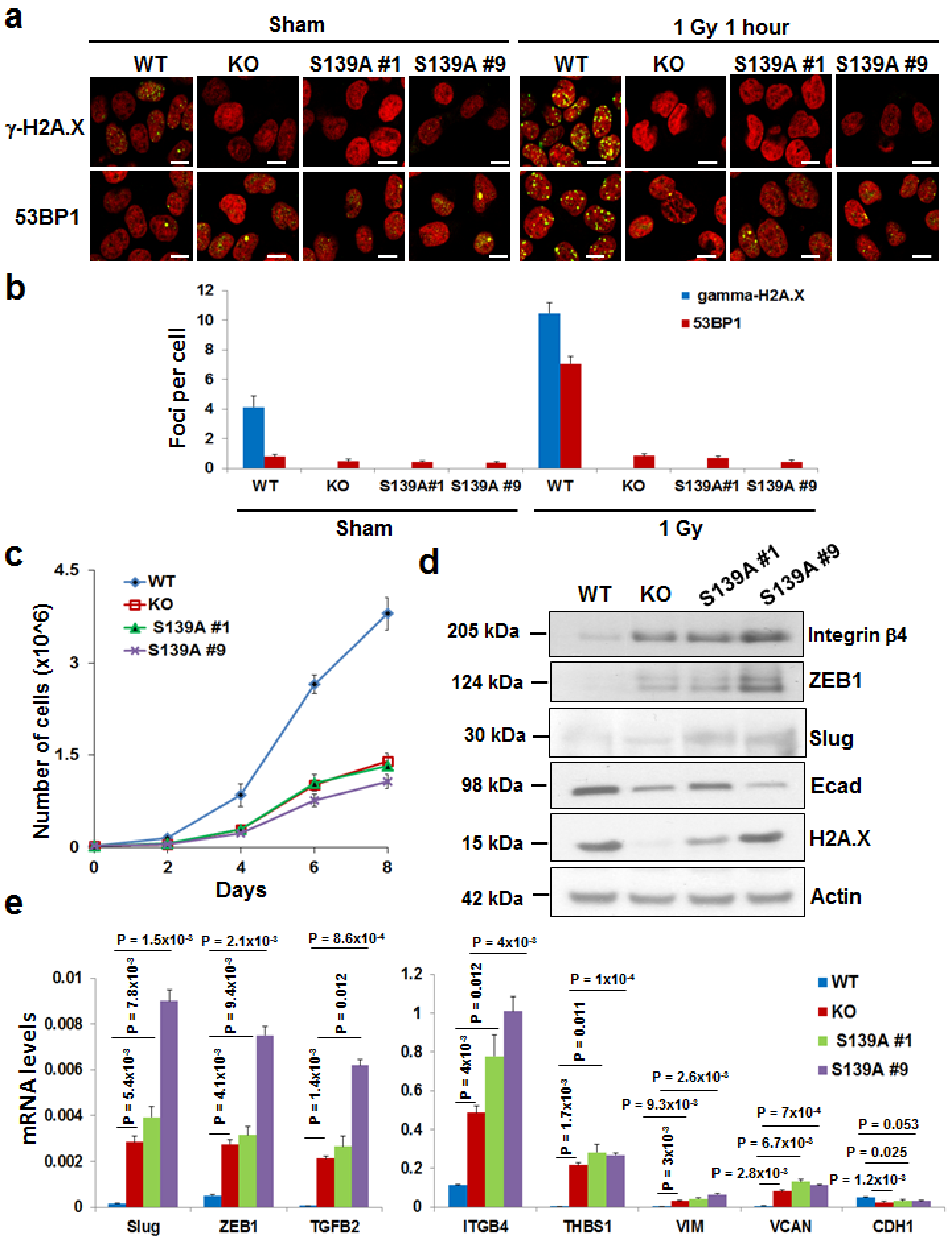

Supplementary figure 11: H2A.X-S139A mutant and EMT. (**a, b**). HCT116 parental cells (WT), H2A.X knockout cells (KO) and two independent clones of H2A.X knockout cells expressing mutant

H2A.X (S139A #1 and S139A #9) were analyzed for their ability to detect DNA damage. Cells were exposed to 1 Gy of ionizing radiation. DNA damage levels were analyzed by counting γ-H2A.X and 53BP1 foci (green) in nuclei counterstained for DNA (red). (**a**) Representative images, Scale bars, 20 μm. (**b**) Quantitation of γ-H2A.X and 53BP1 foci per cell. The foci number is an average of foci per cell in a total of 50 cells per sample. (**c**) Quantification of the proliferation rate of cells described in (a,b). Error bars indicate the S.D. (n = 3). (**d**) Western blot analysis of EMT markers in cells described in (a-c), utilizing actin as a loading control. (**e**) Cells were analyzed for EMT markers transcripts levels. The transcript levels were analyzed by real time PCR. Expression values are relative fold change for genes transcripts of Slug, ZEB1, TGFB2, ITGB4, THBS1, VIM, VCAN and CDH1. Error bars indicate the S.D. (n=3). Statistical significance was determined by a two-tailed, unpaired Student's *t*-test.

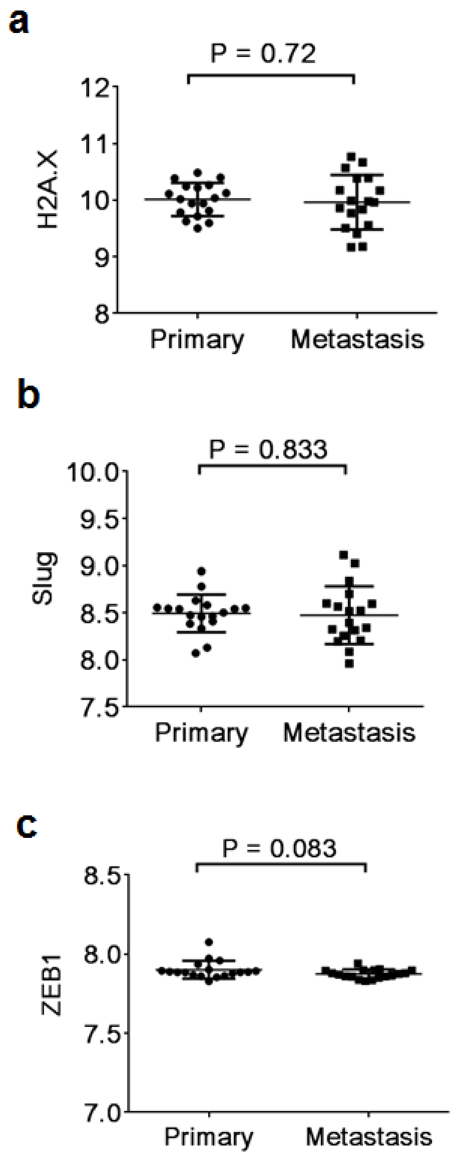

Supplementary figure 12: **H2A.X and Slug /ZEB1 expression in primary tumors and liver metastases** (**a-c**) Transcript level comparison of Slug (**a**), H2A.X (**b**) and ZEB1 (**c**) on a panel of 18 primary colorectal cancers and 18 matched liver metastases, using publicly available datasets from Gene Expression Omnibus, GEO (*GSE14297)*. RNA was hybridized on Human Sentrix-6 V2 (Illumina).Statistical significance was determined by a two-tailed, unpaired Student's *t*-test.

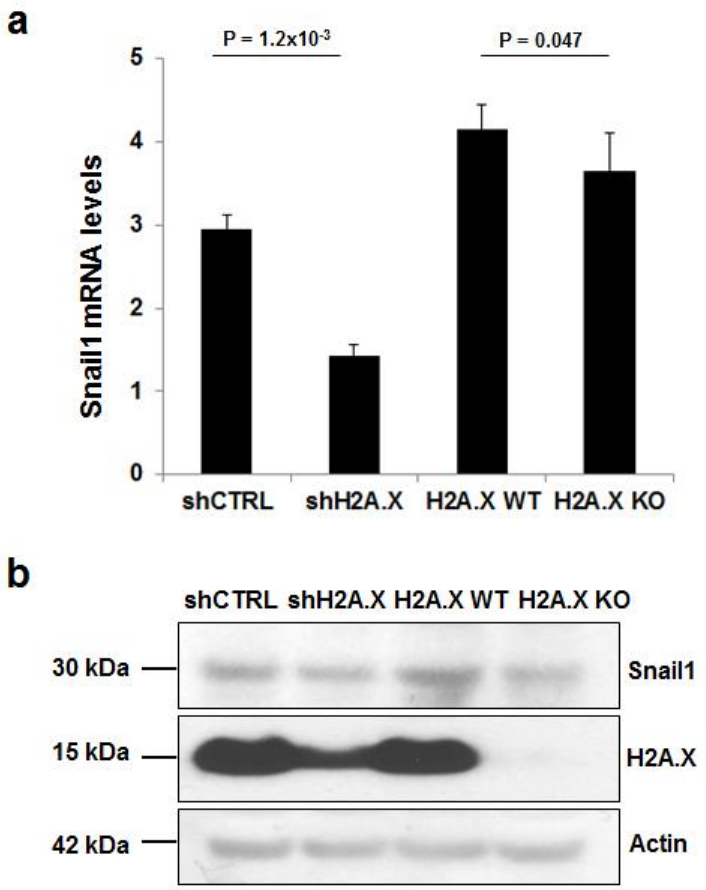

Supplementary figure 13: **Snail1 detection in HCT116 cells depleted for H2A.X.** (**a**) Control cells (shCTRL) and cells silenced for H2A.X (shH2A.X) or parental cells (H2A.X WT) and H2A.X knockout cells (H2A.X KO) were analyzed for Snail1 expression by real time PCR. Expression values are relative fold change for Snail1 normalized to GAPDH, RPLP0 and HPRT1. Error bars represent the S.D. (n = 3). Statistical significance was determined by a two-tailed, unpaired Student's t-test. Cells used in (a) were analyzed for Snail1 expression by western blotting (**b**). Actin was used as loading control.

Fig. 1a

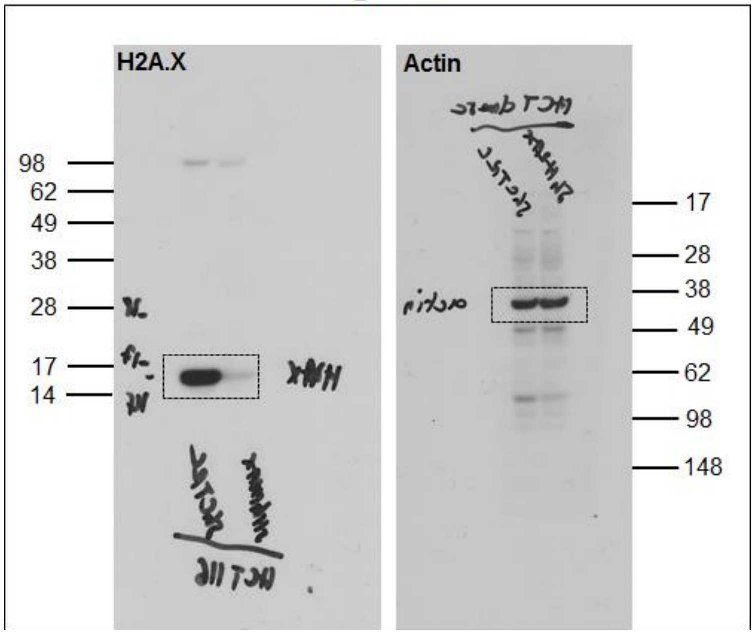
H2A.X
Actin
98
62
49
38
28
17
14
17
28
38
49
62
98
148

Fig. 2a

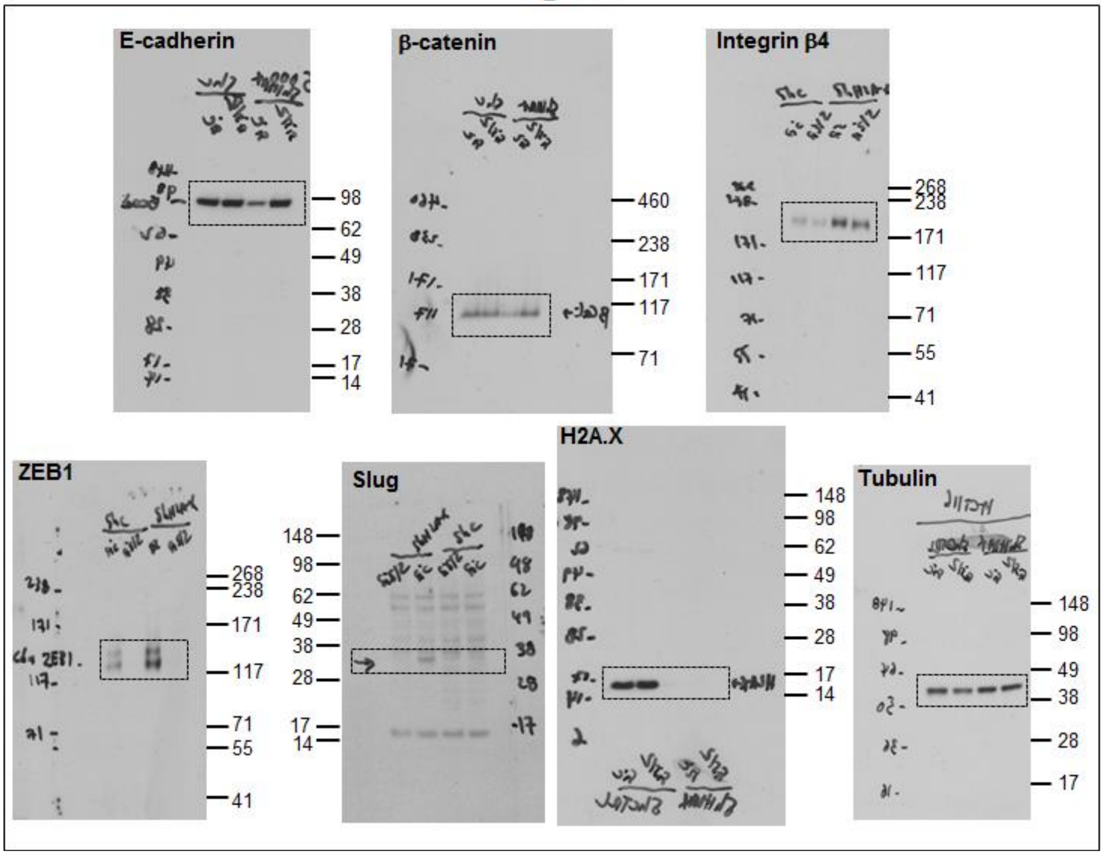
E-cadherin
98
62
49
38
28
17
14
β-catenin
460
238
171
117
71
Integrin β4
268
238
171
117
71
55
41
ZEB1
268
238
171
117
71
55
41
Slug
148
98
62
49
38
28
17
14
H2A.X
148
98
62
49
38
28
17
14
Tubulin
148
98
49
38
28
17

Fig. 2d

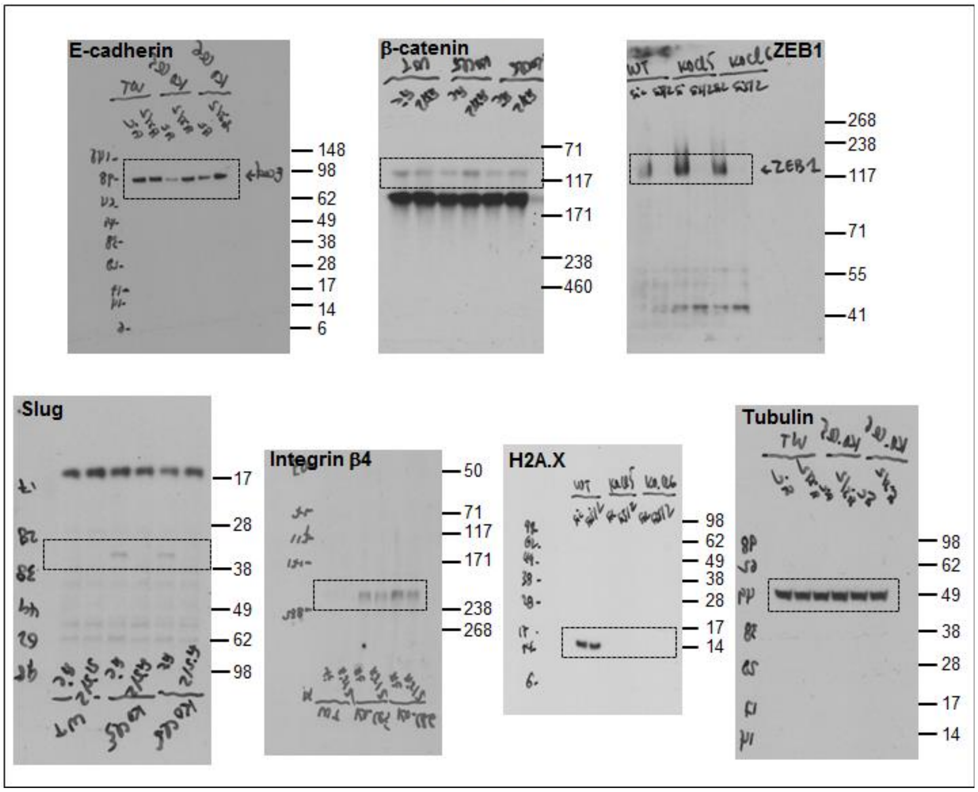
E-cadherin
148
98
62
49
38
28
17
14
6
β-catenin
71
117
171
238
460
ZEB1
268
238
117
71
55
41
Slug
17
28
38
49
62
98
Integrin β4
50
71
117
171
238
268
H2A.X
98
62
49
38
28
17
14
Tubulin
98
62
49
38
28
17
14

Fig. 4a

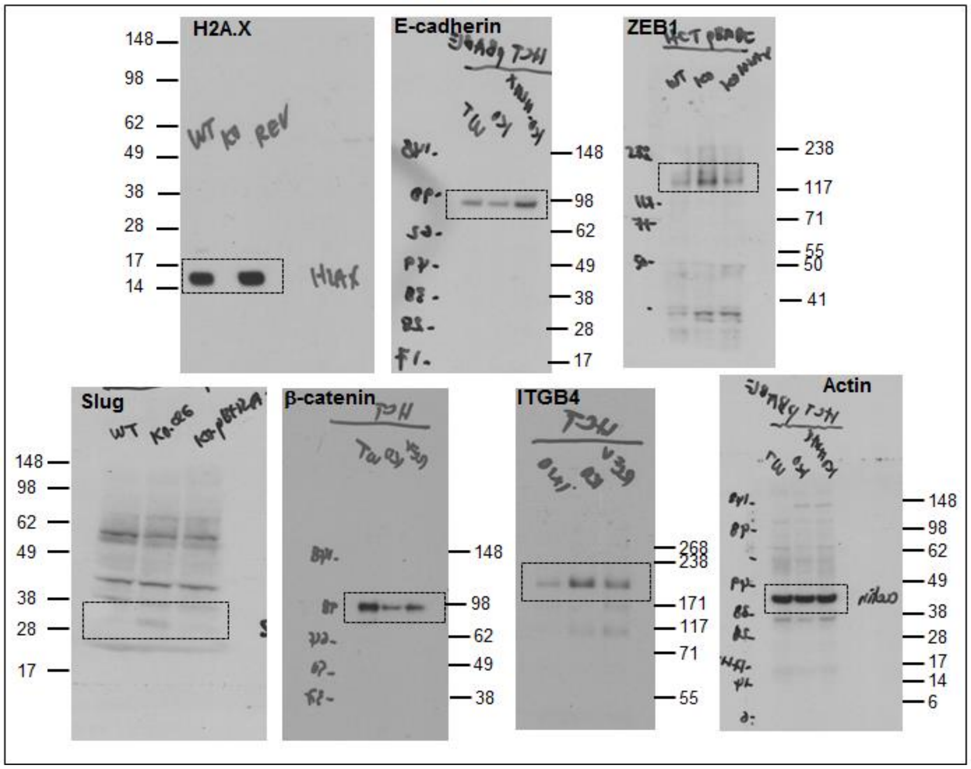
H2A.X
148
98
62
49
38
28
17
14
E-cadherin
148
98
62
49
38
28
17
ZEB1
238
117
71
55
50
41
Slug
148
98
62
49
38
28
17
β-catenin
148
98
62
49
38
ITGB4
268
238
171
117
71
55
Actin
148
98
62
49
38
28
17
14
6

Suppl. Fig. 1b

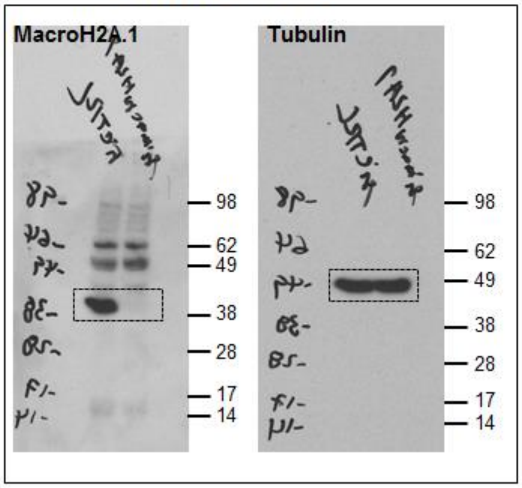

Suppl. Fig. 1d

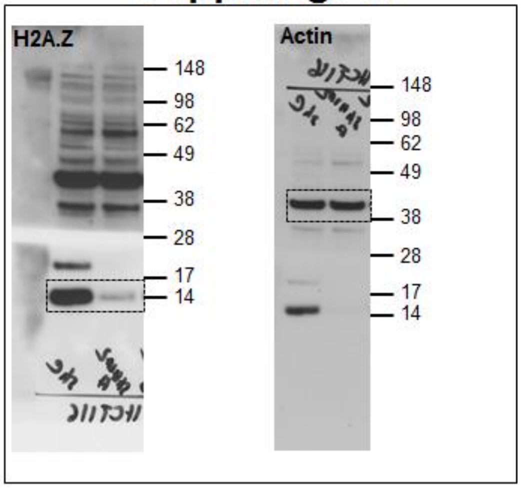

Suppl. Fig. 2a

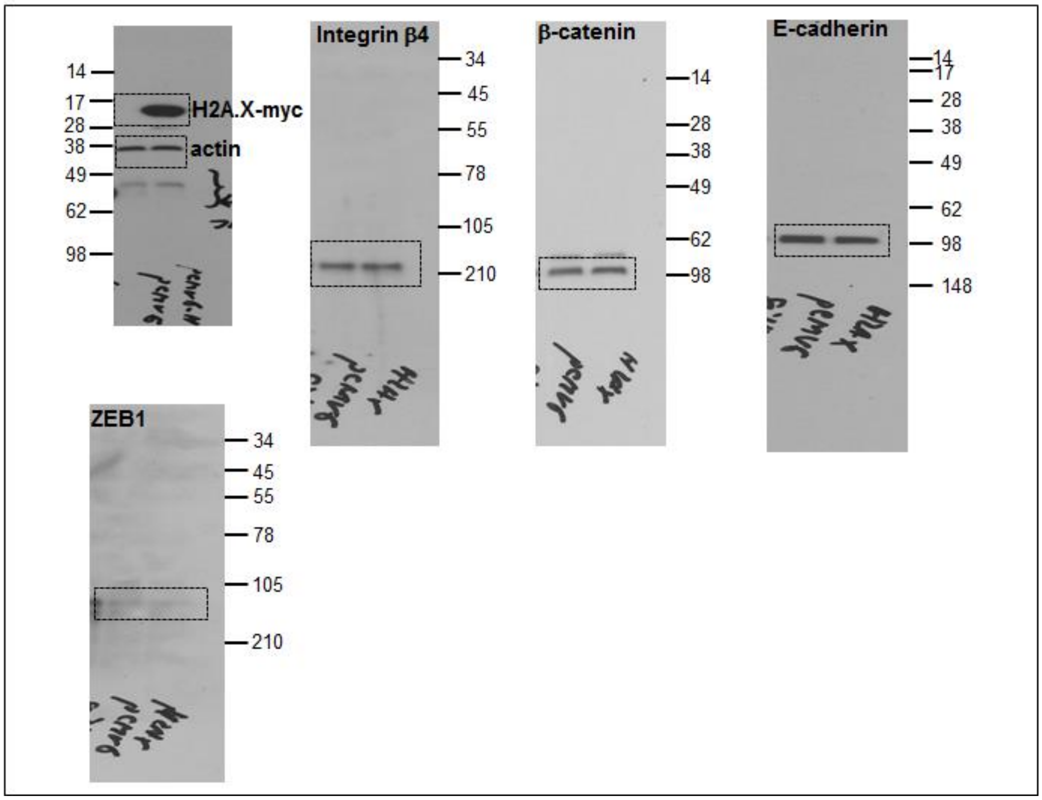

Suppl. Fig. 2c

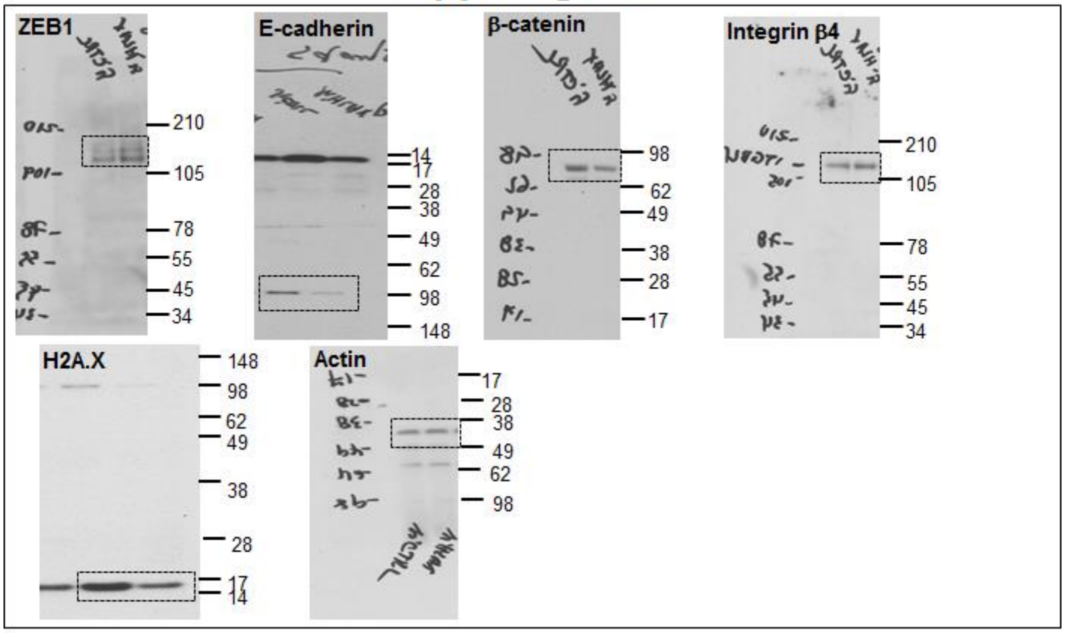

Suppl. Fig. 6c

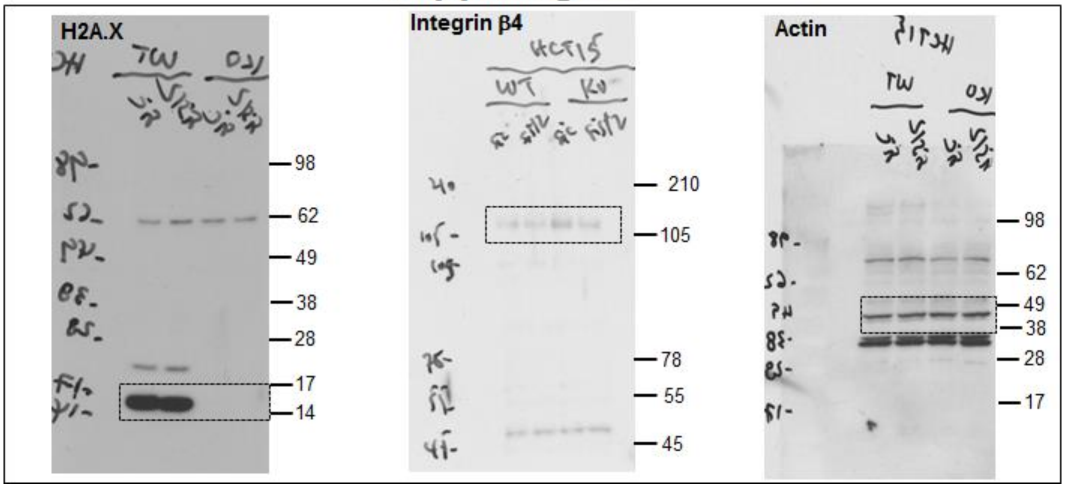

Suppl. Fig. 7a

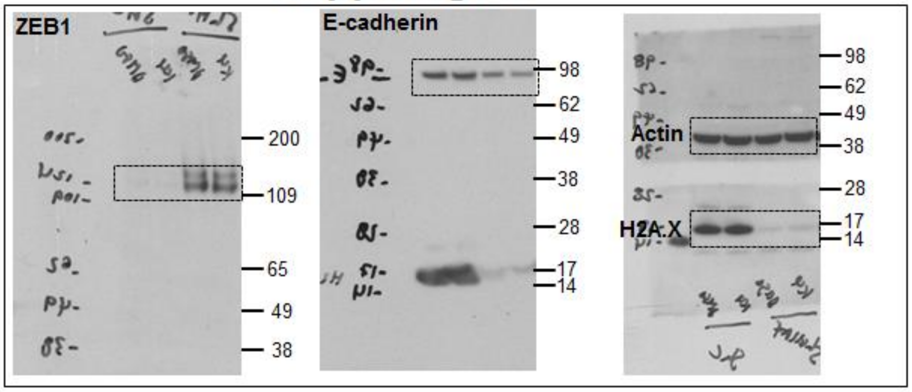

Suppl. Fig. 8b

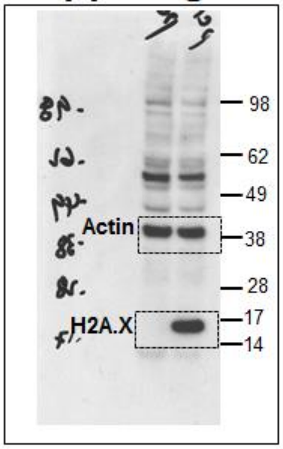

Suppl. Fig. 9c

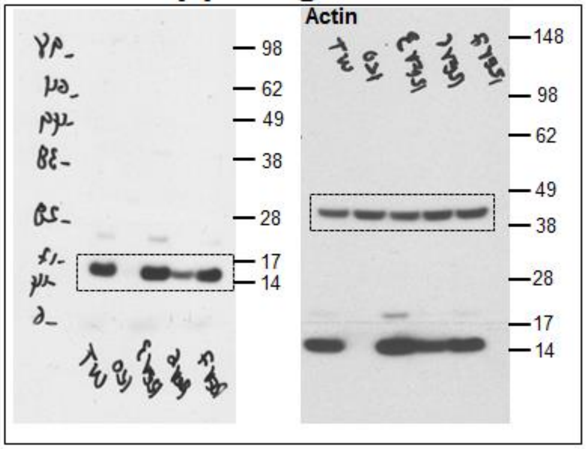

Suppl. Fig. 11d

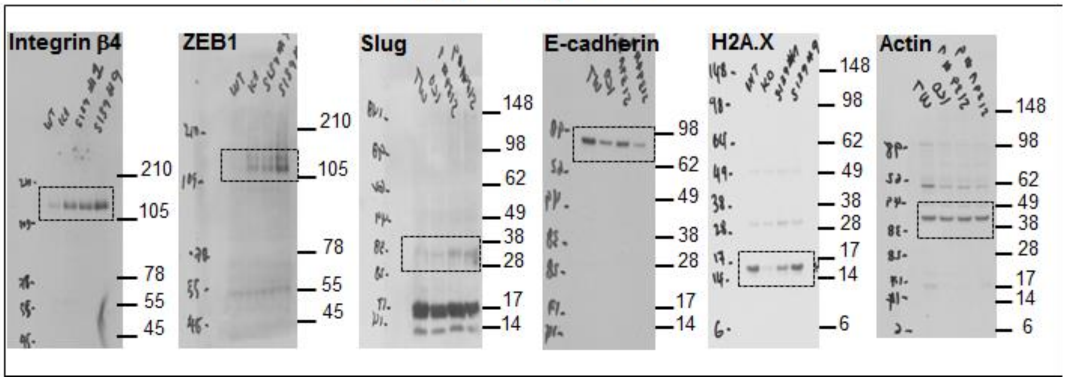

Suppl. Fig. 13b

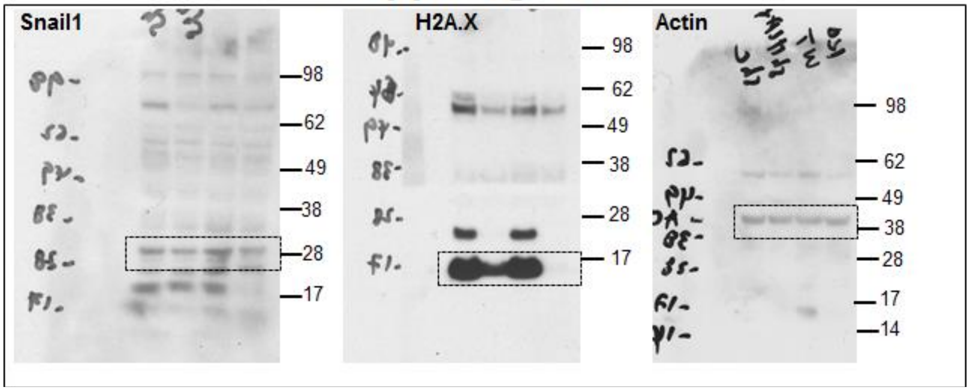

Supplementary Figure 14: Uncropped scans of western blot results displayed in this study

Pool Catalog Number/Dharmacon	siRNA Catalog number	siRNA Name	Target Sequence
D-001810-10	D-001810-01	**ON-TARGETplus Non-targeting siRNA #1**	UGGUUUACAUGUCGACUAA
	D-001810-02	**ON-TARGETplus Non-targeting siRNA #2**	UGGUUUACAUGUUGUGUGA
	D-001810-03	**ON-TARGETplus Non-targeting siRNA #3**	UGGUUUACAUGUUUUCUGA
	D-001810-04	**ON-TARGETplus Non-targeting siRNA #4**	UGGUUUACAUGUUUCCUA
L-011682-00-0005	J-011682-08	**ON-TARGETplus Human H2AFX SMARTpool siRNA #1**	GGGACGAAGCACUUGGUAA
	J-011682-09	**ON-TARGETplus Human H2AFX SMARTpool siRNA #2**	CGACUAGAACCUUAGGCAU
	J-011682-10	**ON-TARGETplus Human H2AFX SMARTpool siRNA #3**	GGAAAGAGCUGAGCCGCUU
	J-011682-11	**ON-TARGETplus Human H2AFX SMARTpool siRNA #4**	GAACUGGAAUUCUGCAGCU
L-011964-00-0005	J-011964-05	**ON-TARGETplus Human H2AFY SMARTpool siRNA #1**	CCAACUAGGCUGAGCAAUG
	J-011964-06	**ON-TARGETplus Human H2AFY SMARTpool siRNA #2**	GUGAUCCACUGUAAUAGUC
	J-011964-07	**ON-TARGETplus Human H2AFY SMARTpool siRNA #3**	CCGAGUUGCUAGCGAAGAA
	J-011964-08	**ON-TARGETplus Human H2AFY SMARTpool siRNA #4**	UGGAAUACCUGACAGCGGA
L-017386-00-0005	J-017386-05	**ON-TARGETplus Human SNAI2 SMARTpool siRNA #1**	UCUCUCCUCUUUCCGGAUA
	J-017386-06	**ON-TARGETplus Human SNAI2 SMARTpool siRNA #2**	GCGAUGCCCAGUCUAGAAA
	J-017386-07	**ON-TARGETplus Human SNAI2 SMARTpool siRNA #3**	ACAGCGAACUGGACACACA
	J-017386-08	**ON-TARGETplus Human SNAI2 SMARTpool siRNA #4**	GAAUGUCUCUCCUGCACAA
L-006564-01-0005	J-006564-10	**ON-TARGETplus Human ZEB1 SMARTpool siRNA #1**	CUGUAAGAGAGAAGCGGAA

	J-006564-11	**ON-TARGETplus Human ZEB1 SMARTpool siRNA #2**	CUGAAAUCCUCUCGAAUGA
	J-006564-12	**ON-TARGETplus Human ZEB1 SMARTpool siRNA #3**	GCGCAAUAACGUUACAAAU
	J-006564-13	**ON-TARGETplus Human ZEB1 SMARTpool siRNA #4**	GCAACAGGGAGAAUUAUUA

Supplementary Table 1: List of siRNAs sequences used in the study. SiRNAs were pre-designed and validated for their efficacy by the manufacturer (Dharmacon, Thermo Scientific, Hudson, NH, USA). ON-TARGET plus Non-targeting siRNA are control siRNAs. ON-TARGET plus Human H2AFX SMART pool refers to siRNA targeting H2A.X, and ON-TARGET plus Human H2AFY SMART pool refers to siRNA targeting MacroH2A.1.
